# Reliability-Aware Cross-Modal Learning Behavior Sensing for Student Cognitive Bias Recognition and Teaching-Oriented Psychological Risk Warning

**DOI:** 10.3390/s26165286

**Published:** 2026-08-20

**Authors:** Luo Xu, Chenlu Jiang, Moxian Lin, Yan Zhan

**Affiliations:** 1 Hong Kong Baptist University, Hong Kong, China; 2 China Agricultural University, Beijing 100083, China; 3 Peking University, Beijing 100871, China

**Keywords:** artificial intelligence-driven sensing, multimodal learning behavior sensing, reliability-aware cross-modal fusion, temporal behavior modeling, educational data mining

## Abstract

With the development of smart classrooms and digital learning platforms, multimodal learning behavior data provide a new sensing basis for understanding students’ cognitive states and psychological risk warnings. However, existing educational data mining methods mainly focus on performance prediction, dropout warning, or surface-level emotion recognition, while continuous and interpretable modeling of deeper cognitive biases and related psychological risks remains insufficient. To address this issue, we propose MLBS-Net, a multimodal learning behavior sensing network for teaching feedback that jointly models students’ textual expressions, behavioral sequences, classroom interactions, and psychological auxiliary signals. MLBS-Net integrates theory-guided textual cognitive bias encoding, temporal behavioral state modeling, and reliability-aware cross-modal fusion to capture psychologically interpretable cognitive patterns, characterize dynamic learning-state changes, and adaptively integrate multimodal information according to data quality and task contribution while providing interpretable feedback for teachers. Experimental results show that MLBS-Net achieves a Macro-F1 of 0.855 for cognitive bias recognition and an AUC of 0.891 for psychological risk warning, outperforming traditional machine learning, unimodal deep learning, and standard multimodal methods. Ablation results further support the effectiveness of theory-guided semantic encoding, temporal behavioral modeling, reliability estimation, and multitask learning. These findings demonstrate that MLBS-Net can jointly characterize cognitive biases and potential psychological risks from multisource learning behaviors, providing a feasible approach for learning-state sensing, risk warning, and interpretable teaching support in smart education.

## 1. Introduction

With the rapid development of online education, smart classrooms, and digital learning platforms, large volumes of multimodal learning behavior data—including textual responses, response time, click trajectories, resource access records, interaction frequency, and assessment performance—are continuously generated during the learning process [1]. Beyond conventional learning logs, these data provide rich behavioral signals that reflect students’ cognitive states and psychological changes, creating new opportunities for intelligent educational mental health assessment [2]. Consequently, leveraging learning process data to identify students’ cognitive biases and potential psychological risks at an early stage has become an important research direction in intelligent education [3]. Cognitive bias is widely regarded as a fundamental mechanism underlying the development of psychological risks [4]. According to Beck’s cognitive theory, persistent negative interpretations of learning experiences, such as examination failure or teacher feedback, may gradually develop into cognitive biases including catastrophic thinking, overgeneralization, negative attribution, self-denial, and low self-efficacy [5]. These maladaptive cognitive patterns further contribute to learning anxiety, task avoidance, reduced classroom participation, and accumulated psychological stress [6]. Therefore, timely identification of cognitive biases can provide teachers with interpretable evidence for early psychological intervention before more severe mental health problems emerge [7]. Traditional educational psychological assessment mainly relies on questionnaires, interviews, and teacher observations, which are typically low-frequency, subjective, and unable to continuously capture fine-grained behavioral changes during learning [8]. Meanwhile, existing educational data mining studies have primarily focused on performance prediction, dropout warning, and learning engagement assessment [9], emphasizing whether students are likely to perform poorly while paying limited attention to the cognitive mechanisms that lead to such outcomes [10]. As a result, existing approaches remain insufficient for continuous, interpretable, and early identification of students’ cognitive biases and psychological risks.

In recent years, deep learning methods have provided new technical pathways for student learning behavior analysis and psychological risk identification [11]. Text models such as BERT, RoBERTa, and MacBERT can extract semantic information from students’ open-ended responses, learning reflections, and discussion forum posts. Temporal models such as long short-term memory (LSTM), gated recurrent units (GRUs), temporal convolutional networks (TCNs), and Transformer can model dynamic behaviors such as login frequency, assignment submission, response time, and resource access paths [12]. Multimodal learning methods can further integrate text, behavior, interaction, and scale information to characterize students’ states from multiple perspectives [13]. However, several limitations remain in existing methods. Many models focus only on emotional polarity or risk classification results, with insufficient support from cognitive psychological theory. Some multimodal methods adopt simple feature concatenation, making it difficult to handle heterogeneity, missingness, and noise among different modalities [14]. In addition, although some models achieve favorable prediction performance, their outputs lack teacher-understandable explanations and are difficult to transform into teaching feedback and intervention suggestions. Zhao et al. [15] proposed an Intelligent Sensing Framework and a Multimodal Framework Transformer, in which classroom video and audio modalities were fused to recognize student behaviors. Gao et al. [16] proposed a unified end-to-end student behavior analysis and feedback framework for physical education classes, in which IMU motion signals were transformed into teaching reports through an MD AR AQA model and a large language model. Che et al. [17] proposed a PPO-based hybrid cognitive language reinforcement learning framework, in which T5 semantic embeddings and cognitive behavioral feedback were integrated to learn adaptive teaching strategies. Zhou et al. [18] proposed a multimodal image-recognition-based behavior analysis system for university smart classrooms. Speech-image and facial-image features were constructed, and feature-level and decision-level fusion algorithms were designed to improve student behavior recognition.

To address the above issues, a multimodal learning behavior sensing network for teaching feedback is proposed in this study for student cognitive bias recognition and early psychological risk warning. The main contributions of this study are summarized as follows. It should be emphasized that the architectural novelty of MLBS-Net does not arise from independently employing pretrained encoders, temporal attention, cross-modal attention, or multitask learning. Instead, the key formulation explicitly couples sample-specific modality reliability with task relevance in the computation of fusion weights. For each modality, reliability is estimated jointly from its representation and explicit quality cues, including missingness, temporal continuity, noise level, and data coverage. This reliability score multiplicatively modulates the task-relevance attention score before normalization over the available modalities. Consequently, a modality that is semantically informative but unreliable in the current sample is prevented from receiving an excessively high fusion weight solely because of its task relevance, distinguishing the formulation from conventional attention or gating based primarily on feature representations. Existing experiments support the additional benefit of this design: the AUC for psychological risk warning increases from 0.851 with the standard Multimodal Transformer to 0.891 with MLBS-Net, while removing reliability estimation or quality-cue input reduces the AUC to 0.861 and 0.858, respectively. These results indicate that the improvement is associated with quality-conditioned reliability modulation rather than merely with combining standard components.

1. A theory-guided learning-behavior-sensing paradigm for cognitive bias recognition is developed. Unlike existing educational AI approaches that mainly focus on learning performance, engagement, or surface-level emotion classification, this study adopts Beck’s cognitive theory as the primary theoretical basis and incorporates attribution theory and self-efficacy theory to construct psychologically interpretable cognitive bias representations. Textual expressions and multisource learning behaviors are thereby mapped beyond general state signals to specific cognitive patterns, such as catastrophic thinking, negative attribution, and low self-efficacy, extending educational prediction from outcome-oriented assessment toward cognitive-mechanism recognition.
2. A temporal and reliability-aware multimodal modeling approach is proposed for uncertain real-world teaching data. In contrast to simple feature concatenation or conventional multimodal methods that mainly capture cross-modal correlations, MLBS-Net jointly models textual semantics, continuous learning behaviors, classroom interactions, and psychological auxiliary information by integrating temporal behavioral state modeling with reliability-aware cross-modal fusion. Modality contributions are adaptively adjusted according to data quality and task relevance, reducing the influence of missing, noisy, and inconsistent observations on cognitive bias recognition and psychological risk warning.
3. An integrated framework connecting cognitive bias recognition, psychological risk warning, and teaching feedback is established. Unlike predictive models that only output performance, emotion, or risk categories, MLBS-Net uses multitask learning to jointly optimize cognitive bias recognition and psychological risk warning through shared cognitive-behavioral representations, and further transforms the predictions into cognitive bias evidence, risk information, and teacher-oriented suggestions. The framework therefore provides not only risk predictions but also interpretable indications of their potential cognitive sources, improving its practical value for intelligent teaching support.

The remainder of this paper is organized as follows. Section 2 reviews the related work relevant to this study. Section 3 describes the data collection and processing procedures and presents the proposed MLBS-Net framework. Section 4 reports the experimental results and discusses the model performance and contributions of its key components. Finally, Section 5 summarizes the main conclusions, limitations, and future research directions.

## 2. Related Work

### 2.1. Learning Behavior Sensing and Educational Data Mining

The basic principle of learning behavior sensing is to regard continuous interaction records generated by students in digital learning environments as behavioral sensing signals. Through the collection, modeling, and analysis of these signals, students’ learning states, engagement levels, and potential problems can be inferred [19]. With the widespread adoption of learning management systems, smart classroom platforms, and online education tools, students’ learning processes can be recorded at a finer granularity, including resource access, click trajectories, page dwell time, video viewing progress, assignment submission, quiz performance, discussion forum posts, and classroom interactions [20]. These data are not isolated operational traces, but external behavioral manifestations of students’ cognitive engagement, learning strategies, and psychological states [21]. Therefore, learning behavior sensing provides a new data foundation for understanding students’ learning processes and enables educational research to gradually shift from outcome-oriented performance analysis to process-oriented state recognition [22]. Educational data mining is commonly used to discover relationships between students’ learning performance and behavioral patterns based on the above learning behavior data [23]. Traditional methods mostly rely on manually constructed features, such as login frequency, click frequency, response duration, submission delay, and quiz scores, and are combined with models such as Logistic Regression, support vector machine (SVM), Random Forest, or XGBoost for performance prediction, learning engagement assessment, dropout warning, and personalized recommendation [24]. In recent years, with the development of deep learning, models such as LSTM, GRU, TCNN, and Transformer have been used to model student behavior sequences and capture dynamic patterns of learning states over time [25]. Attention mechanisms have also been introduced into educational data analysis to identify key learning stages that have a substantial influence on learning performance [26]. However, most existing studies still focus on observable learning outcomes, such as whether students will experience performance decline, whether dropout may occur, or whether engagement is insufficient, while the interpretation of students’ deep cognitive biases and psychological risk formation mechanisms remains limited [27]. Different from these studies, learning behavior is not merely regarded as an auxiliary feature for performance prediction in this study, but is understood as an external sensing manifestation of students’ psychological and cognitive states. Potential cognitive biases and psychological risks are identified from learning rhythm, task completion, interaction changes, and behavioral abnormalities [28].

### 2.2. Cognitive Bias Theories and Mental Health Risk Detection

The core principle of cognitive bias theory is that an individual’s emotional and behavioral responses to external events are not determined entirely by the events themselves, but are influenced by internal cognitive processing patterns, automatic thoughts, and core beliefs [29]. According to Beck’s cognitive theory, when events are interpreted in a long-term negative, absolutized, or imbalanced manner, stable cognitive biases are likely to be formed, which may further induce anxiety, depression, avoidance, and low self-evaluation. In educational contexts, when students face examination failure, teacher evaluation, peer comparison, and learning pressure, biases such as catastrophic thinking, overgeneralization, selective negative attention, self-denial, and black-and-white thinking may occur [30]. For example, a single test failure may be interpreted by a student as permanent inability to learn well, while a single instance of teacher criticism may be understood as evidence that the teacher believes the student lacks ability [31]. These expressions are not merely ordinary negative emotions, but reflect students’ cognitive interpretations of learning events [32]. Reliance on Beck’s cognitive theory alone is insufficient to fully explain changes in learning psychology within educational scenarios; therefore, attribution theory and self-efficacy theory are often adopted as important complementary frameworks [33]. Attribution theory emphasizes how students explain the causes of success or failure. When failure is attributed to internal, stable, and uncontrollable factors, helplessness and avoidance behaviors are more likely to be generated [34]. Self-efficacy theory focuses on students’ judgments of their own learning ability and task completion capability. Low self-efficacy is usually closely associated with decreased learning engagement, challenge avoidance, and increased psychological stress [35]. Existing mental health recognition studies have often been conducted based on questionnaires, interviews, social media text, speech, facial expressions, or physiological sensor data for emotion recognition, depression detection, and stress prediction [36]. Deep learning models can extract complex features from text, speech, visual signals, or physiological signals, but these methods are mostly designed for general mental health scenarios and are less frequently integrated with learning tasks, response behaviors, and classroom interactions in real teaching processes [37]. Therefore, cognitive bias identification within learning scenarios is emphasized in this study, rather than simply equating student psychological risk with general emotion classification [38]. The focus is placed on how students interpret learning failure, feedback pressure, and task challenges, as well as how these cognitive patterns are jointly manifested through textual expressions and learning behaviors [39].

### 2.3. Multimodal Deep Learning for Intelligent Educational Feedback

The basic principle of multimodal deep learning is to obtain more comprehensive and stable representations than those derived from a single modality by fusing data from different sources, structures, and semantic levels [40]. In educational scenarios, students’ psychological and cognitive states often cannot be accurately judged using only one type of data [41]. Textual responses can reflect students’ linguistic interpretations of learning events, behavioral sequences can indicate changes in learning rhythm and task engagement, classroom interactions can reflect participation willingness and social learning states, and psychological scales can provide staged supervisory signals [42]. Therefore, multimodal learning provides a more complete technical pathway for student cognitive bias recognition and psychological risk early warning [43]. In recent years, methods such as multimodal Transformer, cross-modal attention, contrastive learning, and multitask learning have been widely applied to emotion recognition, behavior understanding, medical diagnosis, and educational analysis [44]. In intelligent education, multimodal models can simultaneously analyze students’ linguistic expressions, learning paths, response behaviors, and classroom participation states, thereby providing teachers with more comprehensive student profiles [45]. However, several limitations remain in existing methods. The sampling frequency, data structure, and semantic level vary considerably across modalities, and simple concatenation can easily introduce noise. Mental health-related labels are difficult to obtain, and model training is easily affected by label imbalance and annotation noise. Many models only output classification results and lack explanatory information that can be understood and used by teachers [46]. Especially in student psychological risk recognition tasks, if model outputs cannot be aligned with explicit cognitive theories, it is difficult for teachers to determine the source of risk or formulate specific intervention strategies [47]. Based on this, a multimodal learning behavior sensing framework for cognitive bias recognition is further proposed in this study on the basis of existing multimodal learning research. Through theory-guided textual semantic encoding, temporal behavioral modeling, and reliability-aware cross-modal fusion, student cognitive bias types, psychological risk levels, and teaching feedback suggestions are jointly modeled, enabling the model to provide not only prediction capability, but also educational interpretability and practical teaching support value [48]. Beyond predictive performance and interpretability, algorithmic fairness is an important consideration in educational AI. Even when demographic or other sensitive attributes are not directly used as model inputs, correlated linguistic expressions, learning behaviors, and course-performance variables may act as proxies and induce indirect group dependence in model predictions [49]. Fairness therefore cannot be guaranteed merely by excluding sensitive variables; statistical dependence between predictions and sensitive or proxy attributes should also be examined. Recent work has introduced differentiable mutual-information surrogates as regularization terms for classification, combined with uncertainty estimation to reduce prediction dependence on sensitive attributes while preserving predictive utility. Such approaches provide a relevant methodological basis for fairness auditing and bias mitigation in educational risk prediction.

In summary, three major gaps remain in existing studies. First, educational data mining generally emphasizes the prediction of learning performance, engagement, or risk outcomes, while paying limited attention to the cognitive mechanisms underlying continuous learning behaviors. Second, psychological-state recognition remains insufficiently integrated with cognitive psychological theories, making it difficult to interpret predictions as psychologically meaningful cognitive biases. Third, existing multimodal methods are vulnerable to modality heterogeneity, missing data, and noise, and their outputs often lack teacher-oriented interpretability. To address these limitations, MLBS-Net integrates theory-guided cognitive bias semantic encoding, temporal behavioral state modeling, reliability-aware cross-modal fusion, and multitask learning within a unified framework. This design jointly recognizes cognitive biases and psychological risks while generating interpretable teaching feedback, thereby bridging learning behavior sensing, psychological mechanism interpretation, and practical teaching support.

## 3. Materials and Methods

### 3.1. Data Collection

Data collection in this study was conducted based on a smart-classroom hardware sensing environment and desktop learning behavior acquisition terminals. The collection period was set from 2 September 2025 to 20 December 2025, covering a complete 16-week teaching cycle. The acquisition scenarios included offline smart classrooms, self-study computer laboratories, after-class online learning experimental areas, and network-based learning data in Beijing. To ensure that students’ learning states could be recorded continuously and objectively, textual input, keyboard strokes, mouse clicks, gaze changes, classroom participation, voice interaction, seat check-in, and learning environment conditions during the learning process were uniformly regarded as multisource learning behavior sensing signals, as shown in Table 1. Textual data were mainly recorded synchronously when students completed open-ended responses, learning reflections, and error explanations on learning computers equipped with keyboard–mouse acquisition terminals. Text content, input time, revision frequency, and submission time were stored by the system and used to analyze students’ linguistic expressions regarding learning failure, task pressure, and self-perceived ability. Keyboard and mouse behavioral data were collected using standard USB keyboards and optical mice, respectively. Keystroke intervals, deletion frequency, input pauses, click counts, cursor movement trajectories, and page-switching behaviors were recorded to reflect hesitation during answering, repeated revision, attention fluctuation, and task avoidance tendency. Visual behavioral data were collected using desktop eye trackers and high-definition cameras, mainly including fixation points, fixation duration, revisits, head posture, and facial orientation changes, which were used on reading engagement, attention distraction, and learning fatigue. Classroom interaction data were collected using radio-frequency identification (RFID) check-in devices, classroom response devices, and microphone arrays, including attendance check-in, in-class responses, question asking, discussion participation, and voice interaction frequency. These data were used to characterize students’ classroom participation and active learning willingness. Learning environment data were collected using temperature–humidity, illumination, and noise sensors to control the potential influence of environmental factors on students’ behavioral states and to avoid misclassifying learning-state fluctuations caused by classroom temperature, insufficient illumination, or noise interference as psychological risks. Psychological scale data were collected using tablet terminals in Weeks 1, 6, 11, and 16, covering dimensions such as learning anxiety, learning burnout, self-efficacy, and subjective learning stress. These data were associated with the sensor data according to anonymous student identifiers and timestamps.

A total of 267 students were initially recruited from 5 courses and 13 classes at 3 institutions, involving 20 instructors. Participants were aged 17–25 years, including 131 male and 136 female students; only demographic information permitted by the ethical approval and informed-consent procedure was reported. Inclusion criteria required formal enrollment in the participating courses, participation in the designated learning activities, consent to anonymized learning-behavior collection, and completion of the required staged assessments. Participants were excluded because of voluntary withdrawal, insufficient observation coverage, substantial absence of essential modalities, or unavailable cognitive-bias labels. After these criteria were applied, 243 students were retained and 23 were excluded or withdrew. The overall missing rate across the principal modalities was 89%, with missing observations handled through modality-availability masking and reliability-aware fusion. The cognitive-bias classes contained 25, 21, 19, 16, and 27 effective observations, respectively, while the psychological-risk negative and positive classes contained 169 and 74 effective observations. It should be clarified that these environmental variables are not used as direct input features for cognitive bias recognition or psychological risk prediction in MLBS-Net, nor are they included in the subsequent cross-modal feature fusion. Instead, they provide contextual information during data collection and preprocessing to help identify behavioral fluctuations that may originate from external classroom conditions rather than students’ intrinsic cognitive or psychological states. Specifically, when abnormal temperature or humidity, insufficient illumination, or excessive noise occurs, concurrent decreases in attention, interaction, or response stability may be environmentally induced. The environmental records are therefore used to assist in identifying and screening behavioral segments with evident environmental interference, thereby reducing potential confounding effects. After this procedure, the predictive information used by MLBS-Net consists of textual, learning-behavior, classroom-interaction, and psychological-scale data. Thus, environmental sensing data serve as auxiliary information for data-quality control and interference exclusion rather than as direct predictors of psychological risk.

It should be noted that the “Data volume” values in Table 2 represent the total number of record-level repeated measurements accumulated over the 16-week observation period, rather than the numbers of independent participants or independent samples. A single student can generate numerous keystroke, mouse, eye-tracking, classroom-interaction, and other sensing records across different time points and learning activities. These records were linked using anonymous student identifiers and timestamps and subsequently aggregated into student-level temporal learning representations. Therefore, the sensor-record counts should not be interpreted as the number of independent individuals used for model training. To prevent information leakage caused by repeated measurements, the training, validation, and test sets were partitioned according to student identity, ensuring that records from the same student did not occur across different data subsets. Cognitive-bias labels were defined primarily according to Beck’s cognitive theory, complemented by attribution theory and self-efficacy theory, and a unified annotation guideline was established accordingly. Two annotators with backgrounds in educational psychology independently labeled the de-identified student texts as catastrophizing, overgeneralization, negative attribution, self-denial, or low self-efficacy. Inter-annotator agreement was assessed using Cohen’s κ, yielding κ=0.91. Disagreements were reviewed by a third expert with a background in educational psychology to determine the final labels. All labels were manually checked before model training to avoid relying solely on automated rules or keyword matching, as shown in Table 3.

### 3.2. Data Augmentation

The core principle of data augmentation is to generate new samples with the same supervisory signals by applying reasonable perturbation, rewriting, or masking to the original inputs without changing the true labels and core semantics of the samples. In this way, the training data distribution can be expanded, and the robustness of the model to noise, missingness, and individual differences can be improved. For the student cognitive bias recognition and psychological risk early warning tasks investigated in this study, data augmentation is not simply intended to increase the number of samples. Instead, it is necessary to ensure that the augmented textual expressions, behavioral sequences, and multimodal relationships still reflect the same cognitive bias direction and psychological risk state. Let the original training set be denoted as $D={\left\{\left(x_{i},y_{i}\right)\right\}}_{i=1}^{N}$, where $x_{i}$ denotes the multimodal learning behavior data of the *i*-th student, and $y_{i}$ denotes the corresponding cognitive bias or psychological risk label. The objective of data augmentation is to generate an augmented sample ${\tilde{x}}_{i}$ through a transformation function T(·) while keeping the label unchanged, namely ${\tilde{y}}_{i}=y_{i}$. This process can be formulated as follows:

(1) $${\tilde{x}}_{i}=T\left(x_{i}\right),\;{\tilde{y}}_{i}=y_{i}.$$

For textual augmentation, important cognitive bias signals are often contained in students’ open-ended responses, learning reflections, and discussion forum posts, including negative expressions, absolutized words, failure attribution, and low-ability judgments. Therefore, textual augmentation should follow the principle of psychological semantic consistency. That is, the augmented sentence may differ in expression style, but the cognitive bias category reflected by the original text must not be changed. Let the original text be denoted as $x_{i}^{t}$, the textual augmentation function as $T_{t}\left(\cdot \right)$, and the augmented text as ${\tilde{x}}_{i}^{t}$. The process can be represented as follows:

(2) $${\tilde{x}}_{i}^{t}=T_{t}\left(x_{i}^{t}\right).$$

To avoid label drift, the semantic representations before and after augmentation should remain consistent. If a text encoder $f_{t}\left(\cdot \right)$ is used to obtain vector representations of the original and augmented texts, a semantic consistency constraint can be introduced to ensure that the two representations remain close in the representation space:

(3) $$L_{sem}=1-\frac{f_{t}\left(x_{i}^{t}\right)\cdot f_{t}\left({\tilde{x}}_{i}^{t}\right)}{\left|f_{t}\left(x_{i}^{t}\right)||f_{t}\left({\tilde{x}}_{i}^{t}\right)\right|}.$$

In the specific implementation, synonym rewriting, back-translation, random masking, and psychological keyword-preserving rewriting can be adopted to generate augmented texts. For example, the sentence expressing that poor performance in the current test makes the student feel unsuitable for the course can be rewritten as another sentence indicating that the unsatisfactory score makes the student feel unable to learn the course well, because both expressions indicate negative attribution and low self-efficacy tendencies. However, it should not be rewritten into an expression suggesting that the current poor performance can be improved next time, because such rewriting changes the original cognitive bias direction. For categories such as catastrophic thinking, overgeneralization, self-denial, and low self-efficacy, augmentation strategies must preserve keywords and syntactic structures related to cognitive biases, so that stable psychological semantic patterns rather than ordinary emotional polarity alone can be learned by the model.

For behavioral sequence augmentation, students’ learning logs can be regarded as multichannel temporal sensing signals. Let the behavioral sequence of the *i*-th student be denoted as $X_{i}^{b}\in R^{T\times d}$, where *T* denotes the temporal length, and *d* denotes the behavioral feature dimension, such as the number of clicks, dwell time, response duration, submission delay, and interaction frequency. Since missing logs, uneven sampling, and individual differences in operation habits are frequently observed in real teaching platforms, temporal window cropping, slight temporal perturbation, random masking, and local behavioral replacement are adopted to augment behavioral sequences. Random masking augmentation can be expressed as follows:

(4) $${\tilde{X}}_{i}^{b}=M_{i}⊙X_{i}^{b},$$

where $M_{i}\in {\left\{0,1\right\}}^{T\times d}$ denotes the random masking matrix, and ⊙ denotes element-wise multiplication. When some entries in $M_{i}$ are set to 0, the corresponding behavioral records are masked, thereby simulating the missing click, access, or interaction records that may occur in real teaching systems. For continuous behavioral features, small random perturbations can also be added, so that the model does not rely on a fixed numerical value but pays more attention to behavioral change trends. This process can be formulated as follows:

(5) $${\tilde{X}}_{i}^{b}=X_{i}^{b}+\epsilon ,\;\epsilon ∼N\left(0,\sigma^{2}\right),$$

where ϵ denotes the perturbation term following a Gaussian distribution, and σ controls the perturbation intensity. For the task investigated in this study, the key requirement of behavioral augmentation is to preserve the overall learning state of students. For example, adding slight perturbations to response time is reasonable, but augmenting a student with long-term submission delay into one who consistently submits early would change the underlying characteristics of learning burnout or task avoidance. Therefore, behavioral sequence augmentation should serve the improvement of model robustness without disrupting the true correspondence between cognitive bias and psychological risk. For multimodal augmentation, modality-missing training is emphasized in this study. In real educational scenarios, students may not complete learning reflections every time, and complete interaction records may not be available for every learning task. Therefore, the model should maintain stable prediction when some modalities are missing. Suppose that a student sample contains modalities such as text, behavior, interaction, and scales, denoted as $x_{i}^{t}$, $x_{i}^{b}$, $x_{i}^{u}$, and $x_{i}^{s}$, respectively, with corresponding modal representations $h_{i}^{t}$, $h_{i}^{b}$, $h_{i}^{u}$, and $h_{i}^{s}$. During training, some modalities can be randomly dropped, and a modality mask $m_{k}$ can be used to control whether the *k*-th modality participates in fusion:

(6) $$h_{i}=\sum_{k=1}^{K}m_{k}\alpha_{k}h_{i}^{k},$$

where *K* denotes the number of modalities, $m_{k}\in \left\{0,1\right\}$ denotes modality availability, $\alpha_{k}$ denotes the modality weight learned by the model, and $h_{i}$ denotes the fused multimodal representation. To ensure consistency between the prediction results of augmented and original samples, a cross-modal consistency loss can be further introduced. Let $p_{i}$ denote the prediction distribution of the original sample, and ${\tilde{p}}_{i}$ denote the prediction distribution of the augmented sample. The consistency loss can be defined as follows:

(7) $$L_{con}=D_{KL}\left(p_{i}|{\tilde{p}}_{i}\right).$$

Finally, the model training objective is jointly formed by the classification loss and the augmentation consistency losses:

(8) $$L=L_{cls}+\lambda_{1}L_{sem}+\lambda_{2}L_{con},$$

where $L_{cls}$ denotes the classification loss for cognitive bias recognition and psychological risk prediction, and $\lambda_{1}$ and $\lambda_{2}$ are weighting coefficients. Through the above data augmentation strategies, stable prediction can be maintained by the model under variations in textual expression, behavioral log noise, and incomplete modalities, making it more suitable for deployment in real smart teaching environments. More importantly, the data augmentation strategy in this study is not merely designed for model performance optimization. Instead, it is organized around cognitive bias semantic preservation, learning behavior state preservation, and multimodal risk evidence consistency, so that the augmented samples still conform to educational psychological interpretability and provide a more reliable data foundation for subsequent cognitive bias recognition, psychological risk early warning, and teacher feedback generation.

### 3.3. Proposed Method

#### 3.3.1. Overall

The proposed MLBS-Net starts from aligned and standardized multimodal learning behavior data. The processed student textual input is first fed into the theory-guided textual cognitive bias semantic encoding module. This module is built upon a pretrained language model, in which open-ended responses, learning reflections, error explanations, and discussion texts are encoded into semantic representations. Cognitive bias prompt information constructed based on Beck’s cognitive theory, attribution theory, and self-efficacy theory is further incorporated, enabling the model to focus on expressions related to catastrophic thinking, overgeneralization, negative attribution, self-denial, and low self-efficacy, rather than merely determining whether the emotional polarity of the text is positive or negative. Meanwhile, the processed behavioral sequences are fed into the temporal learning behavior state modeling module. Login frequency, resource access, dwell time, submission delay, response revision frequency, quiz changes, and interaction frequency are regarded as continuous learning behavior sensing signals. Dynamic state representations of students at different learning stages are extracted through a temporal encoder, and key risk segments such as decreased participation, repeated revision, delayed submission, and increased error rates are highlighted through temporal attention. Subsequently, the textual semantic representation, behavioral temporal representation, classroom interaction representation, and psychological scale auxiliary representation are jointly fed into the reliability-aware cross-modal fusion module. Instead of simply concatenating features, this module generates modality reliability weights according to data completeness, stability, noise level, and task relevance across modalities. As a result, information sources can be adaptively adjusted when textual data are missing, classroom interactions are limited, or behavioral logs are incomplete. When risk cues appear simultaneously across multiple modalities, the fused representation enhances the confidence of the final decision. The fused unified representation is further input into a multitask prediction head, through which the possible cognitive bias type of a student and the learning-related psychological risk level are generated simultaneously. Therefore, the model can answer both what type of cognitive bias is exhibited by the student and whether this bias corresponds to a potential risk. Finally, the predicted categories, modality weights, textual evidence, and key behavioral time periods are transformed into teacher-understandable feedback information. In this way, the system not only provides risk labels, but also explains the sources of risk and offers corresponding teaching support suggestions, thereby forming an interpretable early-warning process for smart teaching scenarios.

#### 3.3.2. Theory-Guided Textual Cognitive Bias Semantic Encoding Module

The theory-guided textual cognitive bias semantic encoding module takes the cleaned and standardized student textual representations as input.

**Figure 1:**
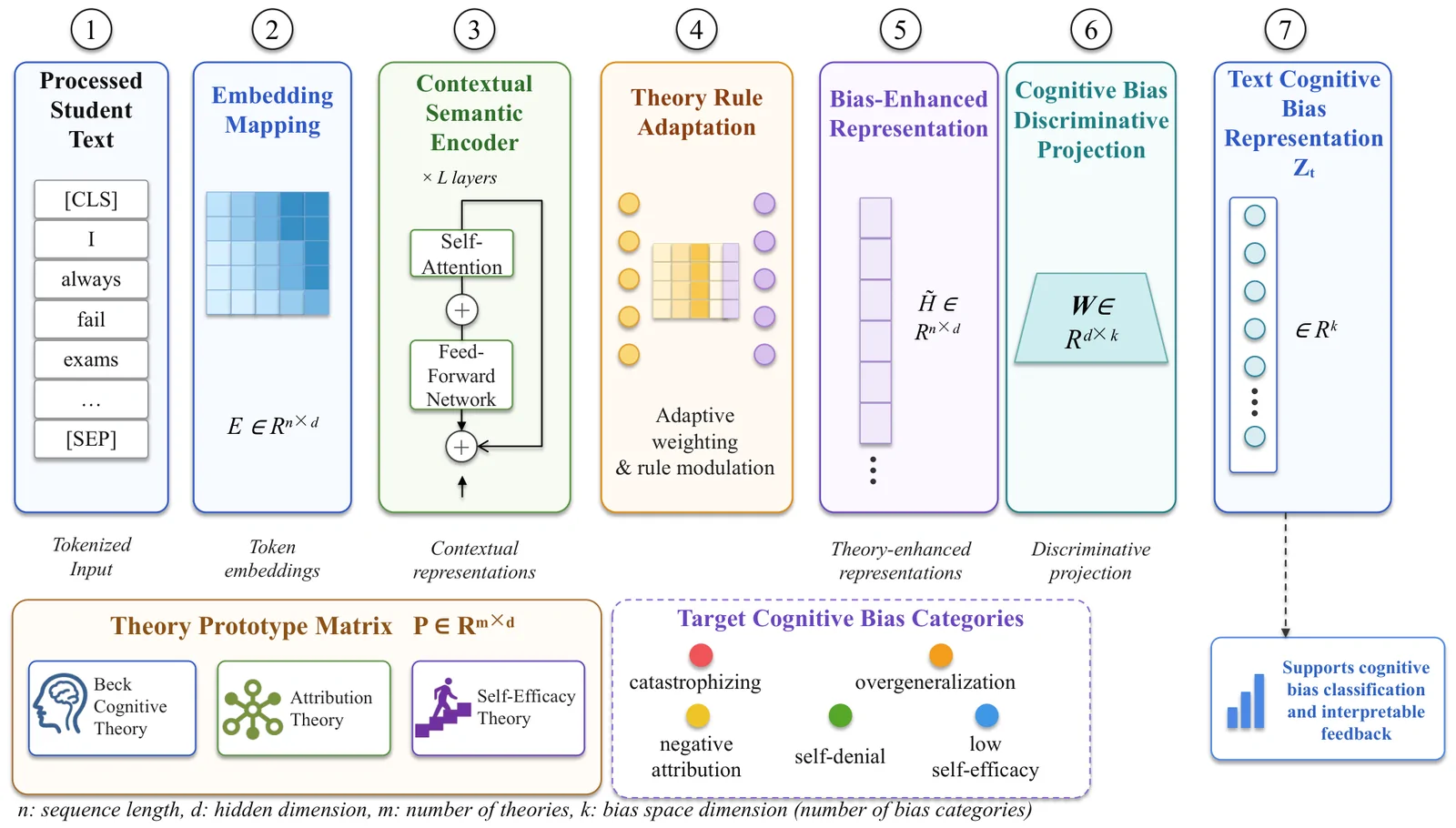
Structure of the theory-guided textual cognitive bias semantic encoding module.

As shown in Figure 1, let the input text tensor be denoted as $X_{t}\in R^{B\times H_{t}\times W_{t}\times C_{t}}$, where *B* denotes the batch size, $H_{t}$ denotes the height of the text sequence, $W_{t}$ denotes the token embedding width, and $C_{t}$ denotes the number of semantic channels. This module consists of an embedding projection layer, $L_{c}$ contextual semantic encoding layers, $L_{r}$ theoretical rule adaptation layers, $L_{d}$ cognitive bias discrimination layers, and an output projection layer. The embedding projection layer first compresses the input into a unified hidden width $D_{t}$. The contextual semantic encoding layer adopts a residual self-attention and feed-forward network structure, with $M_{t}$ attention heads and an intermediate-layer width of $D_{h}$, to capture contextual relationships related to negation, intensity, attribution, and ability judgment in student texts. The encoding process can be written as follows:

(9) $$Q_{t}=X_{t}W_{Q},\;K_{t}=X_{t}W_{K},\;V_{t}=X_{t}W_{V}.$$

(10) $$A_{t}=\mathrm{softmax}\left(\frac{Q_{t}K_{t}^{⊤}}{\sqrt{D_{t}}}\right),\;R_{t}=A_{t}V_{t}.$$

Here, $W_{Q}$, $W_{K}$, and $W_{V}$ are learnable mapping matrices, and $R_{t}$ denotes the context-enhanced textual representation. To introduce psychological theoretical constraints into the model, a theoretical rule prototype matrix $C_{b}\in R^{K\times D_{t}}$ is constructed, where *K* denotes the number of cognitive bias categories, and each prototype corresponds to a cognitive processing pattern defined by Beck’s cognitive theory, attribution theory, and self-efficacy theory. The theoretical rule adaptation layer calculates the matching strength between the textual representation and theoretical prototypes through bilinear interaction:

(11) $$E_{i,k}=R_{t,i}^{⊤}W_{b}C_{b,k},$$

(12) $$\pi_{i,k}=\frac{\exp(E_{i,k})}{\sum_{j=1}^{K}\exp\left(E_{i,j}\right)},$$

where $E_{i,k}$ denotes the correlation between the *i*-th textual segment and the *k*-th cognitive bias prototype, and $\pi_{i,k}$ denotes the theoretical affiliation weight. Subsequently, a bias-enhanced representation is generated according to the theoretical affiliation weights:

(13) $${\hat{R}}_{t,i}=R_{t,i}+\eta \sum_{k=1}^{K}\pi_{i,k}C_{b,k},$$

where η denotes the theoretical enhancement coefficient. This design enables the textual representation not only to retain the original semantics, but also to approach the corresponding cognitive theory space. The cognitive bias discrimination layer further maps the enhanced representation into a global textual representation:

(14) $$z_{t,i}=\mathrm{Norm}\left(W_{o}\cdot \mathrm{MeanPool}\left({\hat{R}}_{t}\right)+b_{o}\right).$$

To ensure clear boundaries among different cognitive bias categories in the representation space, an inter-class margin constraint is introduced:

(15) $$L_{m}=\sum_{i}\sum_{k\neq y_{i}}\max\left(0,\Delta +d\left(z_{i},C_{b,y_{i}}\right)-d\left(z_{i},C_{b,k}\right)\right),$$

where d(·) denotes the distance function, and Δ denotes the class margin. If $L_{m}=0$, the following relation can be obtained:

(16) $$d\left(z_{i},C_{b,y_{i}}\right)+\Delta \leq d\left(z_{i},C_{b,k}\right),\;k\neq y_{i}.$$

This indicates that the distance between the sample representation and the true cognitive bias prototype is at least one margin Δ smaller than the distance to other category prototypes. Therefore, a stable decision boundary can be formed in the theoretical prototype space. The advantage of this module for the present task lies in the fact that surface-level emotional tendencies in texts are not merely learned. Instead, students’ interpretations of learning events are identified through theoretical prototype constraints, allowing general negative expressions to be distinguished from deep cognitive biases such as catastrophic thinking, negative attribution, and low self-efficacy.

#### 3.3.3. Temporal Learning-Behavior-State Modeling Module

The temporal learning behavior state modeling module is used to extract dynamic representations of students’ state changes from continuous learning behavior sensing signals.

**Figure 2:**
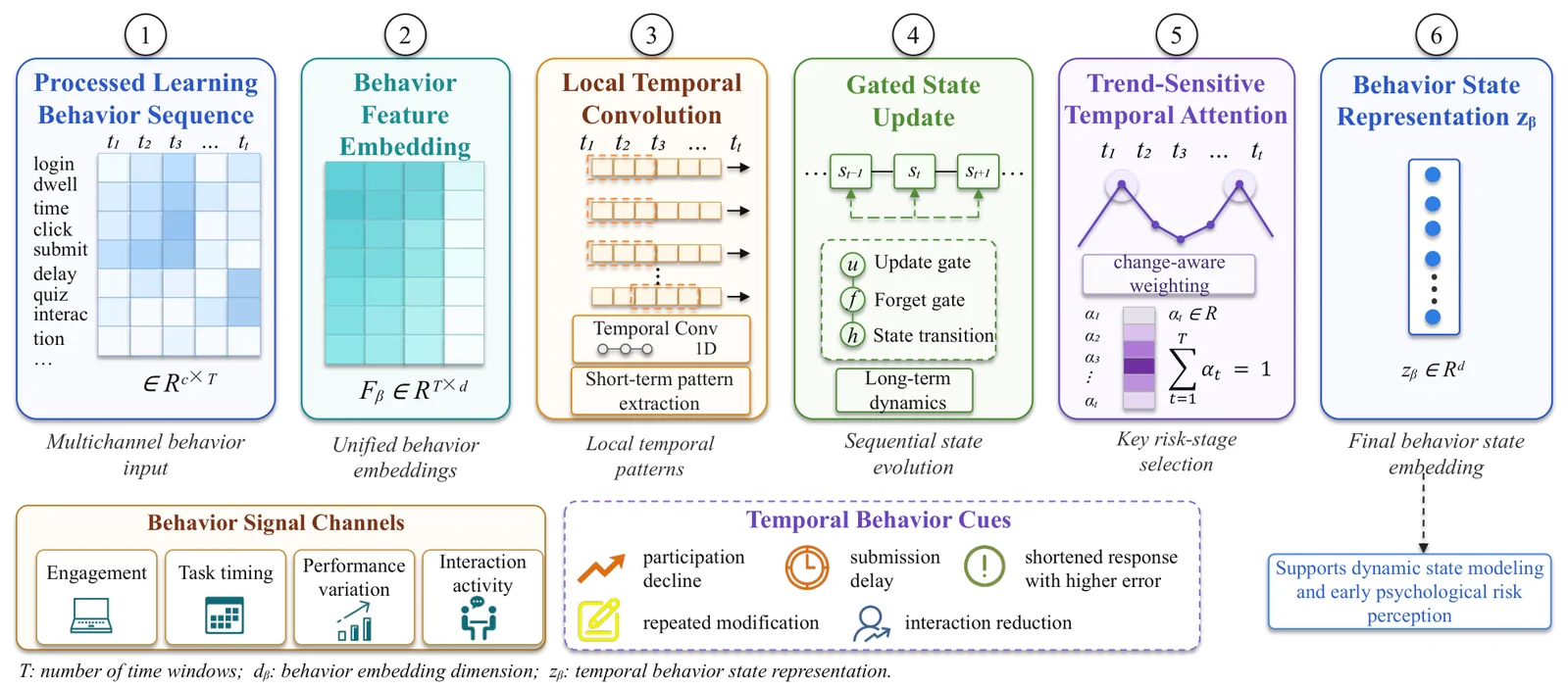
Structure of the temporal learning behavior state modeling module.

As shown in Figure 2, let the processed behavioral input be denoted as $X_{b}\in R^{B\times H_{b}\times W_{b}\times C_{\mathrm{beh}}}$, where *B* denotes the batch size, $H_{b}$ denotes the learning-cycle length or the number of time windows, $W_{b}$ denotes the behavioral feature width within each time window, and $C_{\mathrm{beh}}$ denotes the number of behavioral channels, such as behavioral signals from clicks, dwell time, submissions, quizzes, and interactions. This module consists of a behavioral feature embedding layer, $L_{s}$ local temporal convolution layers, $L_{g}$ gated recurrent state layers, $L_{a}$ trend-sensitive attention layers, and a state output mapping layer. First, the behavioral embedding layer maps multichannel behavioral signals into a unified hidden space, yielding $F_{b}\in R^{B\times H_{b}\times D_{b}}$, where $D_{b}$ denotes the hidden representation width. Subsequently, the local temporal convolution layer adopts one-dimensional causal convolution to extract short-term change patterns between adjacent learning stages. Its kernel height is $K_{b}$, the number of input channels is $C_{\mathrm{beh}}$, and the number of output channels is $D_{b}$, which can be expressed as follows:

(17) $$Y_{t}^{\left(l\right)}=\psi \left(\sum_{r=0}^{K_{b}-1}\Theta_{r}^{\left(l\right)}F_{t-r}^{\left(l-1\right)}+a^{\left(l\right)}\right),\;l=1,\ldots ,L_{s},$$

where $\Theta_{r}^{\left(l\right)}$ denotes the convolution parameter of the *l*-th layer at the *r*-th temporal offset, and ψ(·) denotes a nonlinear activation function. This operation can capture local behavioral patterns such as assignment submission delay, learning-time fluctuation, short-term click anomalies, and changes in quiz response time. To further model the long-term evolution of learning states, a gated recurrent state updating mechanism is introduced after the convolutional output, enabling the model to retain continuous changes in students across time windows:

(18) $$\rho_{t}=\sigma \left(Y_{t}W_{\rho }+s_{t-1}U_{\rho }+c_{\rho }\right),$$

(19) $$\omega_{t}=\sigma \left(Y_{t}W_{\omega }+s_{t-1}U_{\omega }+c_{\omega }\right),$$

(20) $${\bar{s}}_{t}=\tan h\left(Y_{t}W_{s}+\left(\omega_{t}⊙s_{t-1}\right)U_{s}+c_{s}\right),$$

(21) $$s_{t}=\left(1-\rho_{t}\right)⊙s_{t-1}+\rho_{t}⊙{\bar{s}}_{t},$$

where $s_{t}$ denotes the learning state in the *t*-th time window, $\rho_{t}$ controls the writing strength of new behavioral information, and $\omega_{t}$ controls the retention proportion of historical states. This structure is suitable for characterizing dynamic processes such as continuously decreasing participation, gradually increasing task avoidance, or sudden fluctuations in learning engagement. To highlight key risk stages, the trend-sensitive attention layer further calculates the variation magnitude between adjacent states and incorporates it into the weight allocation process:

(22) $$\delta_{t}=\left|s_{t}-s_{t-1}\right|,$$

(23) $$\mu_{t}=\frac{\exp\left(q^{⊤}\tan h\left(W_{\mu }s_{t}+V_{\mu }\delta_{t}\right)\right)}{\sum_{\tau =1}^{H_{b}}\exp\left(q^{⊤}\tan h\left(W_{\mu }s_{\tau }+V_{\mu }\delta_{\tau }\right)\right)}.$$

Finally, the temporal behavioral state representation of a student is obtained by weighting the states of all time windows:

(24) $$z_{b}=\sum_{t=1}^{H_{b}}\mu_{t}s_{t}.$$

The rationality of this attention mechanism can be explained from the perspective of weight monotonicity. For two time windows *p* and *q*, if their basic states are similar, but $\delta_{p}>\delta_{q}$, and $V_{\mu }$ has a positive response to the risk-change direction, then the following relation holds:

(25) $$q^{⊤}\tan h\left(W_{\mu }s_{p}+V_{\mu }\delta_{p}\right)>q^{⊤}\tan h\left(W_{\mu }s_{q}+V_{\mu }\delta_{q}\right).$$

Therefore, the following can be obtained:

(26) $$\mu_{p}>\mu_{q}.$$

This indicates that higher weights are assigned by the model to time windows with more obvious behavioral changes, making early risk signals easier to capture. When applied to the present task, this module does not rely on single test scores or static statistical features. Instead, patterns of state evolution are identified from long-term student learning behavior trajectories. Continuous decreases in login frequency, increased submission delay, reduced interaction, and abnormal quiz changes can be uniformly modeled as temporal sensing signals and can form complementary evidence with subsequent textual cognitive bias representations. As a result, psychological risk early warning becomes earlier and more stable, which is also more consistent with the gradual accumulation of risk in real teaching scenarios.

#### 3.3.4. Reliability-Aware Cross-Modal Fusion and Feedback Module

The reliability-aware cross-modal fusion and feedback module is used to map textual cognitive bias representations, behavioral temporal state representations, classroom interaction representations, and psychological scale auxiliary representations into the same discriminative space. The fusion intensity is dynamically adjusted according to the completeness, stability, and task contribution of different modalities in the current sample.

**Figure 3:**
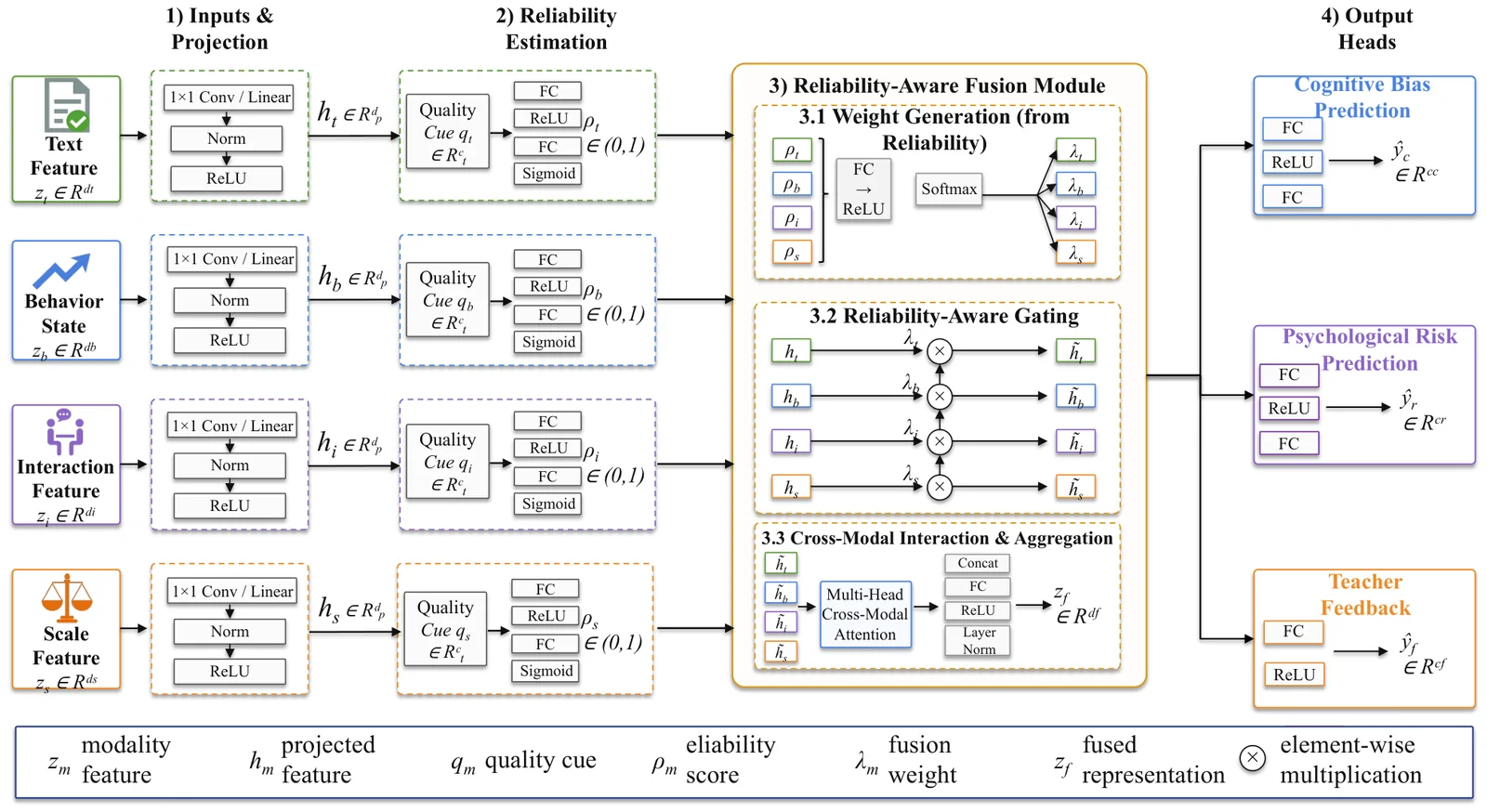
Structure of the reliability-aware cross-modal fusion and feedback module.

As shown in Figure 3, let the four input representations be denoted as $z_{t,i}\in R^{D_{t}}$, $z_{b}\in R^{D_{b}}$, $z_{i}\in R^{D_{i}}$, and $z_{s}\in R^{D_{s}}$, where $D_{t}$, $D_{b}$, $D_{i}$, and $D_{s}$ denote the feature widths of the text, behavior, interaction, and scale modalities, respectively. This module first sets up an $L_{m}$-layer modality projection network. Each layer consists of a linear mapping, a normalization layer, and a nonlinear activation function, through which modality features with different widths are projected into a unified dimension $D_{f}$. For the *m*-th modality, the projection process is expressed as follows:

(27) $$h_{m}^{\left(l\right)}=\mathrm{Norm}\left(\varphi \left(W_{m}^{\left(l\right)}h_{m}^{\left(l-1\right)}+b_{m}^{\left(l\right)}\right)\right),\;l=1,\ldots ,L_{m}.$$

where $h_{m}^{\left(0\right)}=z_{m}$, $W_{m}^{\left(l\right)}\in R^{D_{m}^{\left(l-1\right)}\times D_{m}^{\left(l\right)}}$, and $D_{m}^{\left(l\right)}$ denotes the hidden width of the *l*-th layer. After projection, all modalities are represented as $h_{m}\in R^{D_{f}}$. Subsequently, an $L_{r}$-layer reliability estimation network is constructed. Its inputs include not only the modality representation $h_{m}$, but also the modality quality description vector $q_{m}\in R^{D_{q}}$, such as missing indicators, temporal continuity, noise level, and sample coverage. The reliability score is computed as follows:

(28) $$\rho_{m}=\sigma \left(u_{r}^{⊤}\varphi \left(W_{r}\left[h_{m};q_{m}\right]+b_{r}\right)\right).$$

To simultaneously consider modality reliability and task relevance, a reliability-modulated fusion weight is further designed:

(29) $$\lambda_{m}=\frac{\rho_{m}\exp\left(u_{f}^{⊤}\tan h\left(W_{f}h_{m}+b_{f}\right)\right)}{\sum_{j\in M}\rho_{j}\exp\left(u_{f}^{⊤}\tan h\left(W_{f}h_{j}+b_{f}\right)\right)},$$

where M denotes the set of available modalities, and $\lambda_{m}$ denotes the fusion weight of the *m*-th modality. From the above equation, the following relation can be obtained:

(30) $$\sum_{m\in M}\lambda_{m}=1,\;\lambda_{m}\geq 0.$$

Therefore, the fused representation is a stable convex combination and will not be infinitely amplified due to numerical abnormalities in any single modality. The final cross-modal representation is defined as follows:

(31) $$z_{f}=\mathrm{Norm}\left(\sum_{m\in M}\lambda_{m}h_{m}\right).$$

The robustness of this module can be explained by the weight response relationship. If two modalities *a* and *b* have the same task relevance, but modality *a* has higher reliability, namely $\rho_{a}>\rho_{b}$, then the following relation holds:

(32) $$\frac{\lambda_{a}}{\lambda_{b}}=\frac{\rho_{a}\exp\left(e_{a}\right)}{\rho_{b}\exp\left(e_{b}\right)}.$$

When $e_{a}=e_{b}$, the following can be derived:

(33) $$\frac{\lambda_{a}}{\lambda_{b}}=\frac{\rho_{a}}{\rho_{b}}>1.$$

This indicates that the model can automatically increase the contribution of high-reliability modalities and reduce the influence of missing, noisy, or discontinuous modalities. The fused $z_{f}$ is then fed into an $L_{o}$-layer multitask output network to generate the cognitive bias category distribution and psychological risk level distribution, respectively,

(34) $${\hat{y}}_{c}=\mathrm{softmax}\left(W_{c}z_{f}+b_{c}\right),$$

(35) $${\hat{y}}_{r}=\mathrm{softmax}\left(W_{r}z_{f}+b_{r}\right).$$

The overall optimization objective is formulated as follows:

(36) $$J=J_{c}+\alpha J_{r}+\beta \sum_{m\neq n}{∥\frac{h_{m}}{∥h_{m}{∥}_{2}}-\frac{h_{n}}{∥h_{n}{∥}_{2}}∥}_{2}^{2},$$

where $J_{c}$ denotes the cognitive bias recognition loss, $J_{r}$ denotes the psychological risk prediction loss, and the last term is used to constrain the consistency of different modalities in the shared semantic space. The advantage of this module in the present task lies in its ability to handle common issues in real teaching scenarios, including missing textual data, incomplete behavioral logs, sparse interaction records, and discontinuous scale collection. When textual evidence is sufficient, cognitive bias semantics can be highlighted by the model. When textual information is limited but behavioral trends are obvious, temporal states can be used for risk judgment. When abnormalities occur simultaneously across multiple modalities, early-warning confidence can be improved. Meanwhile, fusion weights, key modalities, and prediction results can be directly transformed into teacher-understandable feedback evidence, enabling the system not only to complete classification and warning, but also to explain risk sources and support teaching intervention.

## 4. Results and Discussion

### 4.1. Experimental Configuration

#### 4.1.1. Hardware and Software Platform

In terms of the hardware platform, all experiments were conducted on a high-performance deep learning workstation equipped with an Intel Xeon Gold series processor, 128 GB of memory, and an NVIDIA RTX 4090 GPU. The GPU contained 24 GB of video memory and was used to accelerate the training processes of the textual encoding, temporal behavioral modeling, and multimodal fusion modules. All experiments were performed under the same hardware environment to ensure fair comparisons between the baseline methods and the proposed model. For traditional machine learning models, feature training and inference were mainly performed on the CPU. For BERT, GRU, Transformer, and the proposed multimodal learning behavior sensing network, model training and parameter updating were performed on the GPU.

In terms of the software platform, the experimental environment was built on the Ubuntu 20.04 operating system, with Python 3.9, PyTorch 2.0, and CUDA 11.8 adopted as the deep learning framework. The Transformers toolkit was used in the text processing stage to load pretrained language models, while jieba, NumPy, Pandas, and scikit-learn were used for text cleaning, feature processing, and evaluation metric calculation. Behavioral sequence modeling, multimodal fusion, and multitask learning modules were implemented based on PyTorch. During the experiments, TensorBoard was used to record training loss, validation performance, and model convergence. Fixed random seeds were adopted to ensure the reproducibility of experimental results.

To prevent information leakage caused by longitudinal repeated measurements from the same student, all dataset partitions were constructed using anonymous student identity as the grouping unit. Textual records, learning behaviors, classroom interactions, psychological scales, and derived temporal samples belonging to the same student were treated as an indivisible group and assigned exclusively to a single data subset. The dataset was first divided into training, validation, and test sets at a student-level ratio of 70%, 15%, and 15%, respectively. Student-level grouped five-fold cross-validation was further employed, in which independent students, rather than individual records, were divided into five mutually exclusive folds. In each run, one student group was held out for testing, while the remaining student groups were used for training and validation. Consequently, no observations from the same student occurred across different partitions, preventing identity-related leakage and ensuring that the reported performance reflects generalization to unseen students.

The detailed training configurations and hyperparameter settings are summarized in Table 4. These configurations were consistently applied to all experiments to ensure fair comparisons among different methods.

The equal-weight configuration for multitask learning was motivated by the complementary roles of cognitive bias recognition and psychological risk prediction. The former characterizes students’ cognitive interpretations of learning events, whereas the latter evaluates potential risks associated with these cognitive and behavioral manifestations. Since no prior evidence indicated that either task should receive a higher optimization priority, an equal weighting strategy was adopted to allow both objectives to contribute equally to shared representation learning. This configuration is consistent with the framework design, where cognitive bias recognition and psychological risk warning are treated as parallel core objectives. The ablation results further demonstrate that removing multitask learning decreases Acc., AUC, and Confidence, indicating that joint supervision provides beneficial shared representations for both cognitive interpretation and risk assessment. More advanced dynamic task-weighting strategies will be investigated in future work.

During model training, an early stopping mechanism was adopted. Training was stopped when the validation Macro-F1 did not improve for 10 consecutive epochs, and the model parameters with the best validation performance were saved. To address class imbalance, inverse-frequency class weights were introduced into the loss function. Specifically, the weight of each class was calculated as $w_{c}=N/\left(C\times n_{c}\right)$, where *N* denotes the total number of training samples, *C* denotes the number of classes, and $n_{c}$ denotes the number of samples in class *c*.

#### 4.1.2. Baseline Models and Evaluation Metrics

Logistic Regression [50], SVM [51], Random Forest [52], XGBoost [53], TextCNN [54], BiLSTM [55], BERT [56], Behavior Transformer [57], Multimodal Transformer [58], and multimodal early warning models (BCL [59] and shi2025 [60]) were selected as comparison methods. Logistic Regression is a linear classification model that can perform cognitive bias or psychological risk prediction based on manually constructed statistical features, with the advantages of a simple structure and strong interpretability. SVM achieves sample separation by maximizing the classification margin and is characterized by stable performance in small-sample and high-dimensional feature scenarios. Random Forest is an ensemble model composed of multiple decision trees, with the advantage of handling nonlinear features and reducing the overfitting risk of a single tree. XGBoost is an ensemble learning model based on gradient boosting and shows strong fitting capability for structured learning behavior features. TextCNN extracts local semantic features from text using convolutional kernels, with the advantages of fast training and suitability for capturing keyword patterns. BiLSTM can model textual or behavioral sequences from both forward and backward directions, enabling contextual dependencies to be captured. BERT encodes textual semantics based on a pretrained Transformer and can obtain deeper semantic representations related to cognitive bias. Behavior Transformer is used to model students’ learning behavior sequences and can capture long-range behavioral dependencies and changes in key learning stages. Multimodal Transformer fuses multimodal information, including text, behavior, and interaction, enabling students’ cognitive states and psychological risks to be comprehensively characterized from multiple perspectives.

Accuracy, Precision, Recall, Macro-F1, AUC, PR-AUC, F1-score, and Confidence were adopted to evaluate model performance in cognitive bias recognition and psychological risk early warning tasks.

(37) $$\begin{array}{cc} Accuracy & =\frac{TP+TN}{TP+TN+FP+FN},\\ Precision & =\frac{TP}{TP+FP},\;Recall=\frac{TP}{TP+FN},\\ F1\text{-}score & =\frac{2\times Precision\times Recall}{Precision+Recall},\\ Macro\text{-}F1 & =\frac{1}{C}\sum_{c=1}^{C}F1_{c},\\ AUC & =\int_{0}^{1}TPR\left(FPR\right)\;dFPR,\\ PR\text{-}AUC & =\int_{0}^{1}Precision\left(Recall\right)\;dRecall,\\ Confidence & =\frac{1}{N}\sum_{i=1}^{N}\max_{c}p_{i,c}, \end{array}$$

where TP, TN, FP, and FN denote true positives, true negatives, false positives, and false negatives, respectively; *C* denotes the number of categories; $F1_{c}$ denotes the F1 value of the *c*-th class; *N* denotes the total number of samples; $p_{i,c}$ denotes the probability that the *i*-th sample is predicted as the *c*-th class by the model; TPR denotes the true positive rate; and FPR denotes the false positive rate. In this study, Confidence is defined as the mean maximum softmax probability across all test samples and is used to describe the model’s average predictive certainty. A higher value indicates that the predicted probability mass is more concentrated on the selected class. However, this metric does not measure probability calibration and should not be interpreted independently as evidence of predictive reliability, since an overconfident model may also produce high maximum softmax probabilities. Confidence is therefore reported only as a supplementary descriptive measure and is interpreted together with the primary task metrics, including Accuracy, Macro-F1, AUC, and PR-AUC.

### 4.2. Cognitive Bias Recognition

This experiment was designed to verify the classification ability of different models in the student cognitive bias recognition task. The focus was placed on whether fine-grained cognitive bias categories, including catastrophic thinking, overgeneralization, negative attribution, self-denial, and low self-efficacy, could be distinguished from students’ textual expressions, learning behaviors, and multimodal learning states.

**Figure 4:**
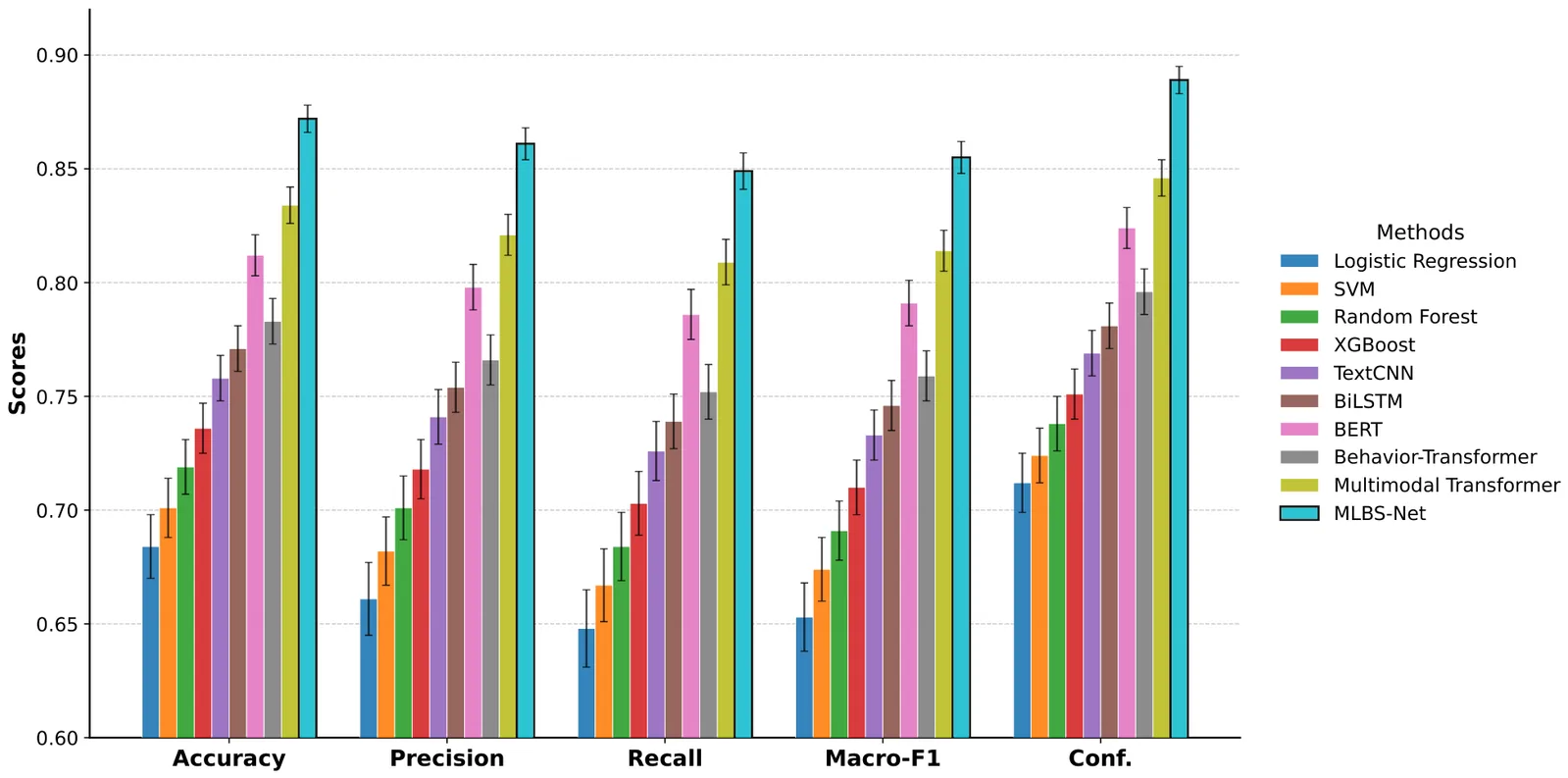
Multi-metric performance comparison of different models for cognitive bias recognition.

As shown in Table 5 and Figure 4, traditional machine learning methods showed relatively limited overall performance. Among them, the Accuracy, Macro-F1, and Confidence of logistic regression were 0.684±0.014, 0.653±0.015, and 0.712±0.013, respectively, indicating that a linear decision boundary is insufficient for characterizing complex semantic relationships in cognitive bias expressions. Compared with logistic regression, SVM achieved a slight improvement, with an Accuracy of 0.701±0.013, suggesting that the margin maximization mechanism can enhance category boundaries. However, SVM still relies on handcrafted features and cannot effectively capture contextual semantics. Random forest and XGBoost achieved Accuracy values of 0.719±0.012 and 0.736±0.011, respectively, outperforming linear models. This improvement can be mainly attributed to the ability of tree-based models to handle nonlinear feature combinations and their higher sensitivity to structured behavioral features such as clicks, dwell time, and revision frequency. However, these models lack deep modeling capability for textual context and psychological semantic relationships; therefore, their Macro-F1 values remained at 0.691±0.013 and 0.710±0.012, respectively. The results of TextCNN and BiLSTM were further improved, with Accuracy values of 0.758±0.010 and 0.771±0.010, respectively, indicating that deep textual models can identify certain cognitive bias cues from local phrases and sequential context. TextCNN is more suitable for capturing keyword combinations within fixed windows, such as absolutized expressions and self-denial phrases, whereas BiLSTM can use bidirectional sequential states to model contextual dependencies. Therefore, BiLSTM slightly outperformed TextCNN in both Recall and Macro-F1.

Further comparison between deep pretrained models and multimodal models shows that BERT achieved Accuracy and Macro-F1 values of 0.812±0.009 and 0.791±0.010, respectively, which were significantly higher than those of TextCNN and BiLSTM. This result indicates that pretrained semantic representations can better understand implicit meanings in student texts. For example, for similar negative expressions, BERT can distinguish general emotional frustration from ability-attribution-related expressions to a certain extent. The Accuracy of Behavior-Transformer was 0.783±0.010, which was lower than that of BERT but higher than those of most traditional models. This suggests that behavioral sequences alone can reflect observable states such as decreased participation, submission delay, and abnormal revisions, but behavioral signals themselves are insufficient for directly explaining specific cognitive bias categories. By fusing textual and behavioral information, Multimodal Transformer improved Accuracy to 0.834±0.008 and Macro-F1 to 0.814±0.009, demonstrating that multimodal complementarity can alleviate the insufficiency of a single modality. The proposed MLBS-Net achieved the best performance, with Accuracy, Precision, Recall, Macro-F1, and Confidence reaching 0.872±0.006, 0.861±0.007, 0.849±0.008, 0.855±0.007, and 0.889±0.006, respectively. From the perspective of model structure, the advantages of MLBS-Net can be attributed to three aspects. First, theory-guided textual encoding enables the representation space to be aligned with cognitive bias theoretical prototypes rather than clustered merely according to emotional polarity, resulting in clearer inter-class boundaries. Second, temporal behavioral modeling supplements learning-state changes that are not explicitly expressed in texts, providing more complete risk evidence. Third, the reliability-aware fusion mechanism dynamically adjusts weights according to modality quality, reducing the interference of noisy modalities in discriminative decisions. Therefore, MLBS-Net achieved the highest performance across all metrics, with narrower confidence intervals, indicating not only stronger classification performance but also higher result stability. Further comparison with domain-specific multimodal approaches shows that BCL and Shi et al. achieve Macro-F1 scores of 0.837 and 0.839, respectively, both exceeding the 0.814 obtained by the generic Multimodal Transformer. This indicates that models specifically designed for student psychological analysis provide stronger representations for fine-grained cognitive-pattern recognition. Nevertheless, MLBS-Net achieves the highest Macro-F1 of 0.855, together with Accuracy, Precision, and Recall of 0.872, 0.861, and 0.849, respectively. These results demonstrate that the advantage of MLBS-Net is maintained against more closely related domain-specific baselines, rather than arising solely from comparison with generic multimodal architectures.

### 4.3. Psychological Risk Early Warning

This experiment was designed to evaluate the early warning capability of different models for students’ learning-related psychological risks. The focus was placed on whether potential risks could be identified from textual expressions, behavioral changes, and multimodal learning states before the risks were clearly manifested as severe learning failure or persistent psychological problems. Unlike cognitive bias recognition, early warning of psychological risk places greater emphasis on the detection of high-risk samples. Therefore, AUC, PR-AUC, Recall, and F1-score are more indicative of the practical value of the model under imbalanced risk samples.

**Figure 5:**
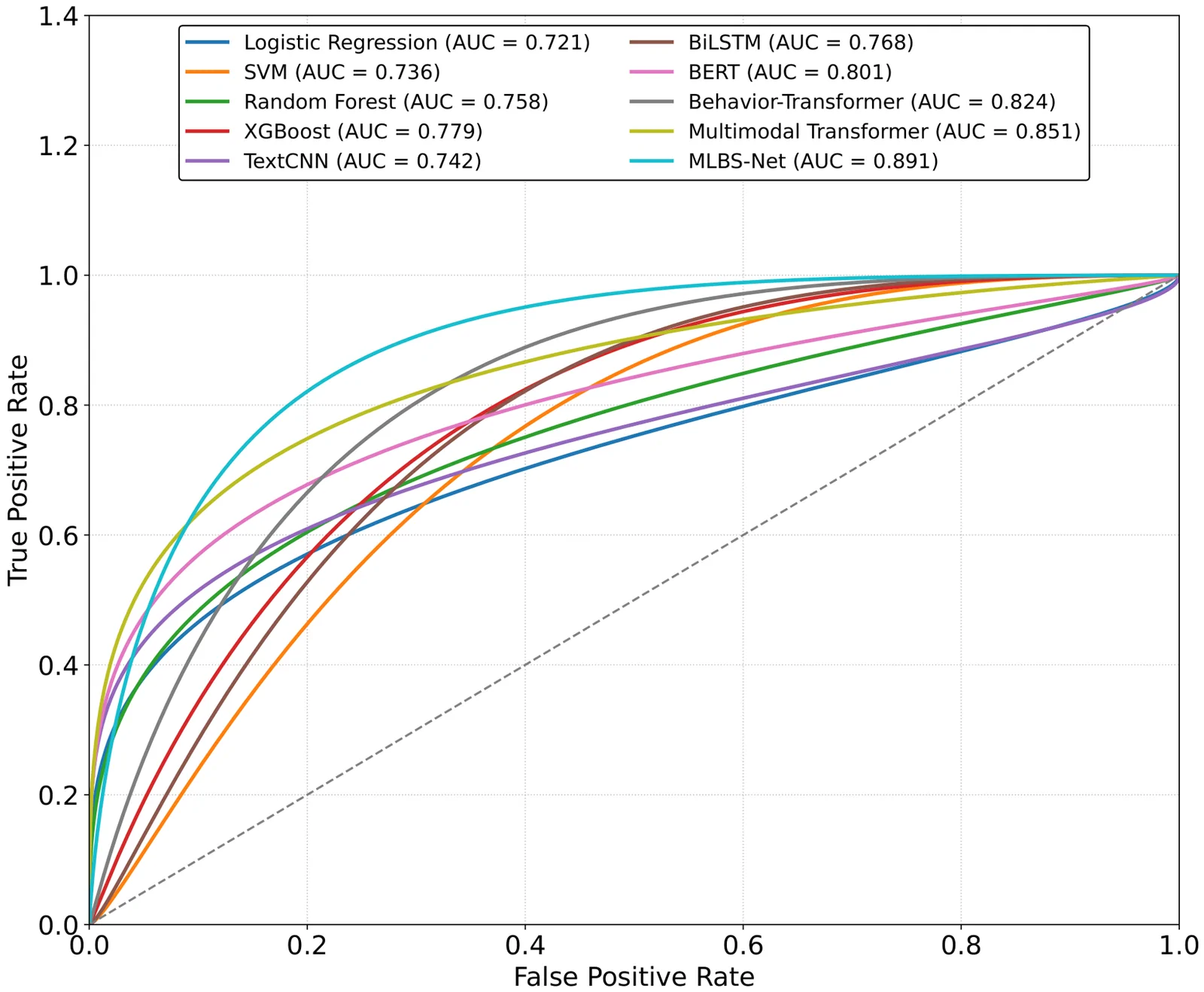
ROC curve comparison of different models for psychological risk early warning.

As shown in Table 6 and Figure 5, the AUC, PR-AUC, Recall, and F1-score of logistic regression were 0.721±0.015, 0.684±0.017, 0.642±0.018, and 0.659±0.016, respectively. These results indicate that a simple linear model can only use part of the explicit statistical features and has insufficient capacity to characterize complex risk formation processes. The AUC of SVM increased to 0.736±0.014, suggesting that the maximum-margin classification mechanism can improve boundary stability. However, it still mainly relies on a fixed feature space and cannot effectively represent the nonlinear accumulation process of psychological risk over time. Random forest and XGBoost achieved AUC values of 0.758±0.013 and 0.779±0.012, respectively, outperforming the above two methods. This indicates that tree-based models can capture nonlinear combinations of features such as assignment delay, decreased interaction, and quiz fluctuation through multilayer feature partitioning. Among them, XGBoost showed better PR-AUC and Recall than random forest because its progressive residual fitting mechanism enables more effective utilization of complex structured behavioral features.

Deep learning models achieved further overall improvements, although their advantages differed across model types. The AUC of TextCNN was 0.742±0.014, which was lower than that of XGBoost, indicating that local textual fragments alone can capture negative expressions but are not sufficiently sensitive to the continuous evolution of psychological risk. BiLSTM achieved AUC and F1-score values of 0.768±0.013 and 0.709±0.014, respectively, outperforming TextCNN mainly because its recurrent structure can preserve contextual dependencies in textual or behavioral sequences. The AUC of BERT reached 0.801±0.011, indicating that pretrained semantic models can more accurately understand implicit psychological cues such as self-denial, low self-efficacy, and negative attribution in student texts. Behavior-Transformer achieved AUC and Recall values of 0.824±0.010 and 0.759±0.013, respectively, which were higher than those of BERT. This suggests that, in the psychological risk early warning task, behavioral trends can better reflect risk accumulation than textual semantics alone, such as continuous decreases in login frequency, increased submission delay, and reduced interaction. By fusing textual and behavioral information, Multimodal Transformer improved AUC to 0.851±0.009, indicating that multimodal complementarity can improve the stability of risk recognition. MLBS-Net achieved the best results, with AUC, PR-AUC, Recall, F1-score, and Confidence reaching 0.891±0.006, 0.867±0.008, 0.828±0.009, 0.841±0.008, and 0.884±0.006, respectively. From the perspective of mathematical modeling characteristics, the advantage of MLBS-Net lies in its simultaneous use of a theory-constrained semantic discriminative space, a temporal state recurrence mechanism, and reliability-aware fusion weights. Thus, deep cognitive biases in texts can be identified, dynamic accumulation of behavioral risk can be captured, and the influence of missing or noisy modalities can be reduced. Therefore, the model improved not only the overall discriminative ability, but also the recall rate and prediction confidence for high-risk students, making it more suitable for early warning in real teaching scenarios. For the domain-specific baselines that more closely reflect educational psychological warning scenarios, BCL and Shi et al. achieve AUC values of 0.872 and 0.874 and PR-AUC values of 0.840 and 0.843, respectively, clearly outperforming the generic Multimodal Transformer. This confirms the effectiveness of domain-oriented multimodal modeling for psychological risk identification. MLBS-Net further improves the AUC and PR-AUC to 0.891 and 0.867 and achieves a Recall of 0.828, compared with 0.807 for BCL and 0.805 for Shi et al. The higher Recall indicates that MLBS-Net identifies a larger proportion of potential high-risk samples while maintaining strong overall discrimination, supporting its practical value for educational risk screening.

### 4.4. Ablation Study

This ablation experiment was designed to verify the actual contribution of each key component in MLBS-Net to the overall performance. The analysis focused on whether theory-guided textual encoding, temporal behavioral modeling, reliability estimation, cross-modal interaction, multitask learning, theory consistency constraints, quality cue input, and the teacher feedback branch jointly constituted the main sources of model performance improvement.

**Figure 6:**
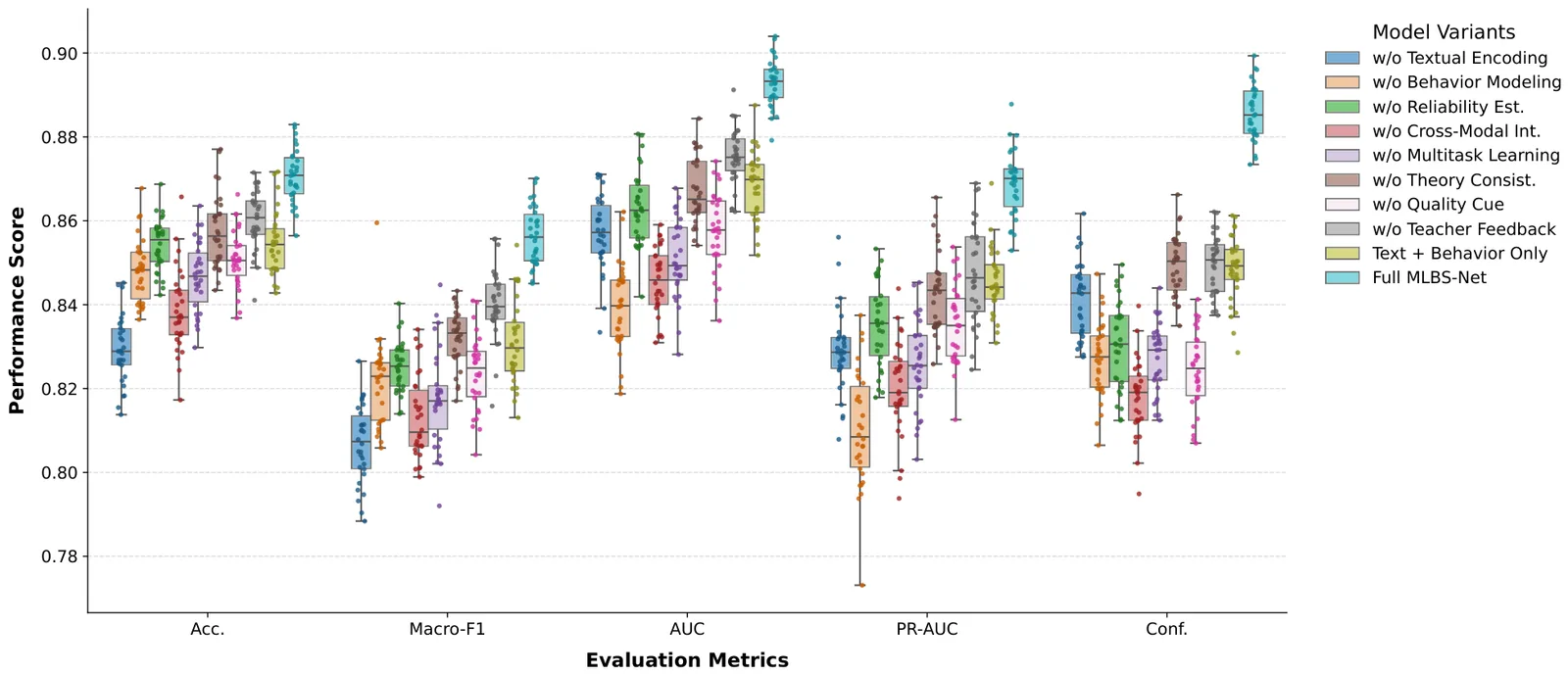
Performance distribution comparison of different MLBS-Net ablation variants.

As shown in Table 7 and Figure 6, after removing theory-guided textual encoding, the Acc. and Macro-F1 of the model decreased to 0.831±0.009 and 0.808±0.010, respectively. This indicates that theory-guided textual cognitive bias encoding plays a core role in cognitive bias recognition, because ordinary textual representations can capture emotional polarity but are insufficient for distinguishing fine-grained cognitive processing patterns such as catastrophic thinking, negative attribution, and low self-efficacy. After removing temporal behavior modeling, AUC and PR-AUC decreased to 0.839±0.010 and 0.812±0.012, respectively, showing a clear performance decline. This suggests that the behavioral temporal module is more critical for psychological risk early warning, because psychological risk is usually manifested as dynamic processes such as continuous decreases in participation, submission delay, and reduced interaction rather than as single static features. After reliability estimation was removed, the model achieved an Acc. of 0.852±0.008, while Conf. decreased to 0.831±0.009. This demonstrates that, when missingness and noise exist in multimodal data, reliability estimation can help reduce the interference of low-quality modalities. After cross-modal interaction was removed, Acc. and Macro-F1 were 0.838±0.009 and 0.815±0.010, respectively, indicating that simple fusion is insufficient for fully establishing the correspondence between textual cognitive biases and behavioral risk manifestations.

Further analysis of other variants shows that, after Multitask Learning was removed, Acc., AUC, and Conf. were 0.844±0.008, 0.852±0.009, and 0.829±0.009, respectively. This indicates that shared information exists between cognitive bias recognition and psychological risk prediction, and that multitask learning enables the model to jointly learn cognitive interpretation patterns and risk-level judgments through collaborative optimization. After theory consistency constraint was removed, the model still maintained relatively good performance, with an Acc. of 0.856±0.008, but the result remained lower than that of the full model. This suggests that the theory consistency constraint is not the only source of performance improvement, but it can further regularize the representation space, making samples of the same cognitive bias category more compact and boundaries between different categories clearer. After quality cue input was removed, Conf. decreased to 0.822±0.009, indicating that modality quality cues have a substantial influence on prediction confidence, because the model loses the basis for assessing text length, behavioral continuity, and interaction sparsity. After the teacher feedback branch was removed, the model still achieved an Acc. of 0.861±0.007, but the performance was lower than that of the full model. This indicates that, although the feedback branch mainly serves explanatory output, its auxiliary supervision can also promote the learning of representations with greater teaching relevance. When only Text + Behavior was used, AUC reached 0.869±0.008, indicating that text and behavior are the two most important modalities. However, without interaction, scale information, and reliability fusion, the performance still did not reach that of the full model. Full MLBS-Net achieved the best results across all metrics, with Acc., Macro-F1, AUC, PR-AUC, and Conf. reaching 0.872±0.006, 0.855±0.007, 0.891±0.006, 0.867±0.008, and 0.884±0.006, respectively. From the perspective of mathematical modeling characteristics, the complete model simultaneously incorporates theoretical prototype constraints, temporal state recurrence, modality reliability weighting, and multitask shared representation learning. Therefore, a more stable discriminative space can be formed, and higher prediction performance and confidence can be maintained under noise, modality missingness, and class imbalance.

### 4.5. Computational Complexity Analysis

To further evaluate the practical applicability of MLBS-Net, a computational complexity analysis was conducted from four perspectives: the number of trainable parameters, floating-point operations (FLOPs), inference time, and GPU memory consumption. Although the proposed framework integrates multiple modules, including theory-guided textual cognitive bias encoding, temporal behavioral state modeling, reliability-aware cross-modal fusion, and multitask prediction, its computational efficiency is important for potential deployment in real smart education platforms. Therefore, the computational costs of MLBS-Net were compared with representative baseline models under the same hardware and software environment. All measurements were obtained using the same test set and averaged over multiple inference runs to reduce measurement fluctuations.

The results in Table 8 demonstrate that MLBS-Net achieves a favorable balance between computational efficiency and modeling capability. Compared with traditional machine learning methods, including Logistic Regression, SVM, Random Forest, and XGBoost, MLBS-Net introduces additional computational overhead due to the joint modeling of textual, behavioral, interaction, and psychological modalities. However, this additional cost is necessary for capturing complex semantic patterns, temporal learning dynamics, and cross-modal relationships that cannot be effectively represented by handcrafted features. Compared with deep learning-based baselines, MLBS-Net maintains a relatively compact architecture. In particular, although BERT and Multimodal Transformer contain 109.84 M and 86.42 M parameters, respectively, MLBS-Net requires only 15.93 M parameters, with 3.576 G FLOPs and 5.847 ms/sample inference time. The GPU memory consumption of MLBS-Net is 1.973 GB, which is substantially lower than that of BERT (4.936 GB) and Multimodal Transformer (5.738 GB). This indicates that the proposed reliability-aware cross-modal fusion mechanism does not introduce excessive computational burden while improving the robustness of multimodal learning behavior sensing. The computational analysis further shows that MLBS-Net is more efficient than conventional Transformer-based multimodal methods while preserving the advantages of multimodal representation learning. Although the proposed framework integrates additional modules, including theory-guided textual encoding, temporal behavioral modeling, and reliability estimation, the overall computational cost remains acceptable for practical smart education applications. These results suggest that MLBS-Net provides a suitable trade-off between predictive performance and deployment efficiency, making it feasible for future intelligent teaching platforms with continuous student-state sensing requirements.

### 4.6. Discussion

The experimental results indicate that the value of MLBS-Net lies not only in improving the classification performance of cognitive bias recognition and psychological risk early warning, but also in transforming subtle signals generated during real learning processes into interpretable teaching feedback. Compared with traditional educational data mining methods, such as Logistic Regression, SVM, Random Forest, and XGBoost, which mainly rely on manually designed behavioral statistics for performance prediction or dropout warning, MLBS-Net focuses on identifying the cognitive mechanisms underlying students’ learning behaviors. By incorporating theory-guided textual cognitive bias encoding based on Beck’s cognitive theory, attribution theory, and self-efficacy theory, the proposed framework can distinguish cognitive patterns such as catastrophic thinking, negative attribution, and low self-efficacy rather than simply detecting negative emotional expressions. Compared with unimodal deep learning approaches, including BERT-based text models and temporal sequence models based on LSTM, GRU, or Transformer, MLBS-Net further benefits from the complementary information among textual expressions, temporal learning behaviors, classroom interactions, and psychological auxiliary signals. Furthermore, compared with conventional multimodal Transformer-based approaches that mainly rely on feature interaction or attention-based fusion, MLBS-Net introduces reliability-aware cross-modal fusion to explicitly consider modality availability and data quality. The experimental results demonstrate that MLBS-Net achieved a psychological risk warning AUC of 0.891, compared with 0.851 achieved by the standard Multimodal Transformer. In addition, removing the reliability estimation module reduced the AUC to 0.861, while removing quality-cue information reduced it to 0.858. These results indicate that the performance improvement is not merely caused by combining multiple modalities, but is mainly associated with adaptive modality weighting according to sample-specific reliability, which is important for practical educational scenarios with missing and noisy observations.

In practical teaching management scenarios, MLBS-Net can provide more fine-grained student state profiles for course instructors, class advisers, and learning support centers. In large-scale classes, blended learning environments, and self-directed learning platforms, teachers usually cannot continuously observe every student’s cognitive and psychological changes. Students’ potential psychological pressure may gradually appear through subtle signals, such as delayed assignment submission, increased response revisions, reduced classroom interaction, and negative self-evaluations in learning reflections. For example, during a 16-week university course, a student may maintain normal quiz performance during the early weeks but later exhibit reduced resource access, delayed assignment completion, decreased classroom participation, and repeated expressions of inability to learn the course well. Traditional performance-based warning systems may only detect problems after significant academic decline, whereas MLBS-Net can identify potential risk patterns earlier by jointly analyzing low self-efficacy expressions and continuous behavioral withdrawal trends. Moreover, the model provides not only a risk prediction but also interpretable evidence regarding possible cognitive sources. For instance, a student who interprets a single poor quiz result as evidence of permanent failure may be identified as exhibiting catastrophic thinking or negative attribution rather than merely insufficient learning engagement. Consequently, teachers can provide more targeted interventions, such as helping students establish achievable short-term goals, reinterpret learning setbacks, and adjust learning strategies. Therefore, MLBS-Net can serve as an auxiliary decision-support tool for smart teaching platforms, learning warning systems, and student support services rather than replacing teachers’ professional judgment.

Although multimodal sensing provides richer information for understanding student states, it does not completely eliminate potential algorithmic bias. Textual expressions, learning rhythms, interaction patterns, and course-related behaviors may still correlate with demographic background, socioeconomic conditions, language proficiency, disability status, course context, or instructor characteristics. Therefore, the model may partially rely on proxy relationships rather than exclusively capturing the intended cognitive constructs. In practical deployment, the predictions generated by MLBS-Net should be regarded as supporting evidence for teacher observation and further communication, rather than as direct criteria for high-stakes educational decisions. Fairness auditing should be conducted to evaluate subgroup performance and investigate potential dependence between predictions and sensitive or proxy attributes. In addition, several limitations should be acknowledged. Although the dataset covers a complete teaching cycle and includes multiple learning behavior modalities, the number of participants remains relatively limited, and further validation on larger and more diverse educational populations is required. Moreover, cognitive bias labels are based on expert annotation guided by psychological theories, which may still involve a certain degree of subjectivity despite achieving high inter-annotator agreement. Future research will investigate larger-scale longitudinal studies, adaptive intervention strategies, causal analysis of learning behavior and psychological states, and fairness-aware optimization methods to further improve the reliability and practical applicability of intelligent educational sensing systems.

### 4.7. Limitations and Future Work

Several limitations remain in this study. First, although the stability of cognitive bias recognition and psychological risk early warning was improved through multimodal learning behavior sensing data, the experimental scenarios were mainly concentrated within a specific course cycle and a relatively fixed smart classroom environment. Student populations, course difficulty, teacher feedback styles, and usage habits of teaching platforms may affect the generalization ability of the model. Second, psychological risk labels were mainly constructed through the integration of staged scales, teacher observations, and behavioral anomaly cues. Although such labels can meet the requirements of teaching warning, they cannot replace professional psychological diagnosis. Therefore, the model output should be understood as a teaching support signal rather than a medical or clinical conclusion. Third, although theory-guided textual encoding and a teacher feedback mechanism were introduced, the feedback content was still mainly based on rule-based generation and evidence matching. Its explanatory ability for complex individual differences, family background, long-term stress sources, and other deeper factors remains limited. Future research can further expand the data scale across schools, courses, and semesters to verify the stability of the model in different educational scenarios. Meanwhile, longer-term longitudinal tracking data can be introduced to analyze the continuous evolution relationships among cognitive bias, learning behavior changes, and psychological risk. In addition, teacher intervention records and subsequent student performance can be incorporated to evaluate whether model feedback can truly improve learning participation, self-efficacy, and psychological stress states, thereby enabling MLBS-Net to develop from a risk recognition tool into a closed-loop smart teaching support system. Because this study involves student behavioral and psychological-risk information, practical deployment must follow strict ethical and privacy principles. Data collection should be based on informed consent, with privacy protected through de-identification, data minimization, secure storage, and controlled access, while applicable data-protection regulations such as the GDPR should be respected. Potential algorithmic bias and the consequences of false-positive warnings, including stigmatization, unnecessary attention, or psychological pressure, should also be considered. MLBS-Net outputs should serve only as auxiliary evidence for teachers and should not automatically trigger punitive or other high-stakes decisions. Teachers should review warnings together with actual observations, while persistent or severe psychological concerns should be referred to qualified mental-health professionals, maintaining a clear boundary between educational support and professional psychological intervention. Another important limitation is that algorithmic fairness across different student populations has not yet been systematically evaluated. Because the current data collection protocol was not designed specifically for protected-attribute fairness analysis, the available sample does not support reliable comparisons across all demographic or other sensitive subgroups. Future studies will, where appropriate consent, privacy protection, and sufficient subgroup sample sizes permit, report subgroup-specific AUC, F1, and Recall and examine statistical dependence between model predictions and sensitive attributes or potential proxies. If meaningful disparities are identified, dependence-mitigation strategies, such as differentiable mutual-information regularization, will be incorporated into the training objective to reduce unintended reliance on non-target group attributes, while explicitly evaluating the trade-off between fairness and predictive performance. In addition, the current psychological risk warning is formulated as a binary classification task and does not further distinguish different levels of risk severity. Future research will extend risk prediction to multi-level identification, such as mild, moderate, and severe risk, when reliable graded annotations become available, and further investigate the correspondence between risk levels and differentiated teaching intervention strategies to provide more fine-grained and targeted support for students with different risk profiles. Furthermore, the current evaluation primarily examines student-level cross-individual generalization and does not yet provide a strict chronological assessment of prospective early-warning performance. Although the 16-week dataset contains timestamped observations and staged psychological assessments in Weeks 1, 6, 11, and 16, future studies should employ rolling-window or landmark-based evaluation. This setting would prevent potential temporal information leakage and more directly determine whether behavioral changes preceding a risk assessment can predict subsequent psychological risk, thereby providing a more rigorous validation of the claimed early-warning capability.

## 5. Conclusions

Despite the rapid development of smart classrooms, online learning platforms, and artificial intelligence-driven educational sensing technologies, several important scientific gaps remain in understanding students’ cognitive and psychological states from learning process data. Existing educational data mining studies have mainly focused on outcome-oriented tasks, such as performance prediction, engagement analysis, and dropout warning, while providing limited understanding of the cognitive mechanisms underlying students’ learning behaviors. In addition, existing psychological state recognition approaches often rely on questionnaires or isolated behavioral signals, making it difficult to achieve continuous, interpretable, and learning-context-aware assessment. Furthermore, conventional multimodal learning methods usually assume that different data sources are complete and equally reliable, which limits their applicability in real teaching environments with missing observations, noise interference, and heterogeneous data quality. Addressing these gaps, this study proposes a multimodal learning behavior sensing network (MLBS-Net) for cognitive bias recognition and learning-related psychological risk early warning, providing a new perspective from outcome prediction toward cognitive mechanism understanding and teaching-oriented support. In the proposed framework, multisource sensing information generated during students’ learning processes, including textual input, keyboard–mouse interaction, classroom interaction, eye-tracking behavior, and staged psychological scales, is jointly modeled. Instead of relying solely on academic performance indicators or questionnaire results, MLBS-Net captures students’ cognitive and psychological changes through the interaction among textual semantics, temporal behavioral patterns, classroom participation, and psychological auxiliary signals. The main contributions include three aspects. First, a theory-guided textual cognitive bias semantic encoding module is developed by incorporating Beck’s cognitive theory, attribution theory, and self-efficacy theory, enabling the model to distinguish ordinary negative expressions from deeper cognitive biases such as catastrophic thinking, negative attribution, and low self-efficacy. Second, a temporal behavioral state modeling module is designed to capture continuous learning-state transitions reflected by behaviors such as decreased participation, delayed submission, repeated revision, and reduced interaction. Third, a reliability-aware cross-modal fusion mechanism is proposed to adaptively adjust modality contributions according to data quality and availability, improving robustness under realistic teaching conditions while generating interpretable feedback for teachers. Experimental results demonstrate that MLBS-Net achieves competitive performance in cognitive bias recognition, with Accuracy, Precision, Recall, Macro-F1, and Confidence reaching 0.872, 0.861, 0.849, 0.855, and 0.889, respectively. For psychological risk early warning, the model achieves an AUC of 0.891, PR-AUC of 0.867, Recall of 0.828, F1-score of 0.841, and Confidence of 0.884, outperforming traditional machine learning methods, unimodal deep learning methods, and standard multimodal Transformer methods. Ablation experiments further verify the effectiveness of theory-guided semantic encoding, temporal behavioral modeling, reliability estimation, and multitask learning. Overall, this study fills the gap between educational data sensing and cognitive psychological interpretation by providing a feasible artificial intelligence-driven framework for continuous learning-state sensing, cognitive bias analysis, and early psychological risk support. The proposed approach may serve as an important foundation for future intelligent education systems that aim to provide personalized, interpretable, and human-centered teaching assistance.

## Figures and Tables

**Table 1:** Statistics of multimodal learning behavior hardware sensing data collection.

Data Type	Hardware Device or Sensor Model	Data Volume
Open-ended response text	Logitech K120 USB Keyboard	18,640
Learning reflection text	Logitech K120 USB Keyboard	12,580
Error explanation text	Logitech K120 USB Keyboard	9360
Keystroke behavior	Logitech K120 USB Keyboard	518,600
Mouse click behavior	Logitech M100r Optical Mouse	624,800
Mouse movement trajectory	Logitech M100r Optical Mouse	486,720
Gaze point trajectory	Tobii Eye Tracker 5	382,400
Fixation duration record	Tobii Eye Tracker 5	296,800
Head posture record	Logitech C920S HD Pro Webcam	214,600
Facial orientation record	Logitech C920S HD Pro Webcam	198,400
Classroom check-in record	MFRC522 RFID Reader	64,320
Classroom voice interaction	ReSpeaker 4-Mic Array	42,680
Classroom temperature and humidity record	Bosch BME280	230,400
Classroom illumination record	BH1750FVI Light Sensor	230,400
Classroom noise record	LM393 Sound Sensor Module	230,400
Psychological scale record	Huawei MatePad SE Tablet	4800

**Table 2:** List of abbreviations used in this paper.

Abbreviation	Full Name
AI	Artificial Intelligence
AUC	Area Under the Receiver Operating Characteristic Curve
BERT	Bidirectional Encoder Representations from Transformers
BiLSTM	Bidirectional Long Short-Term Memory
F1	F1 Score
FC	Fully Connected Layer
FPR	False Positive Rate
GDPR	General Data Protection Regulation
GRU	Gated Recurrent Unit
IMU	Inertial Measurement Unit
LSTM	Long Short-Term Memory
MacBERT	MLMask as Correction BERT
MLBS-Net	Multimodal Learning Behavior Sensing Network
PPO	Proximal Policy Optimization
PR-AUC	Area Under the Precision–Recall Curve
ReLU	Rectified Linear Unit
RFID	Radio-Frequency Identification
ROC	Receiver Operating Characteristic
RoBERTa	Robustly Optimized BERT Pretraining Approach
SVM	Support Vector Machine
T5	Text-to-Text Transfer Transformer
TCN	Temporal Convolutional Network
TCNN	Temporal Convolutional Neural Network
TextCNN	Text Convolutional Neural Network
TPR	True Positive Rate
USB	Universal Serial Bus
XGBoost	Extreme Gradient Boosting

**Table 3:** Main notation used in this paper.

Symbol	Description
D	Original training dataset consisting of multimodal student samples and their corresponding labels.
*N*	Number of student samples in the training dataset.
$x_{i}$, $y_{i}$	Multimodal learning behavior data of the *i*-th student and its corresponding cognitive bias or psychological risk label.
${\tilde{x}}_{i}$, ${\tilde{y}}_{i}$	Augmented sample generated from $x_{i}$ and its corresponding label, respectively.
T(·)	General data augmentation transformation applied to the original sample.
$x_{i}^{t}$, ${\tilde{x}}_{i}^{t}$	Original and augmented textual data of the *i*-th student, respectively.
$T_{t}\left(\cdot \right)$	Textual augmentation function that preserves cognitive-bias-related semantics.
$f_{t}\left(\cdot \right)$	Text encoder used to obtain semantic representations of original and augmented texts.
$L_{sem}$	Semantic consistency loss between the original and augmented textual representations.
$X_{i}^{b}$, ${\tilde{X}}_{i}^{b}$	Original and augmented behavioral sequences of the *i*-th student, respectively.
*T*, *d*	Temporal length of the behavioral sequence and behavioral feature dimension, respectively.
$M_{i}$	Binary random masking matrix used to simulate missing behavioral records.
ϵ, σ	Gaussian perturbation term and its perturbation intensity, respectively.
$x_{i}^{k}$, $h_{i}^{k}$	Input data and learned representation of the *k*-th modality for the *i*-th student.
*K*	Total number of modalities involved in multimodal fusion.
$m_{k}$	Binary modality-availability indicator determining whether the *k*-th modality participates in fusion.
$\alpha_{k}$	Learned fusion weight assigned to the *k*-th modality.
$h_{i}$	Fused multimodal representation of the *i*-th student.
$p_{i}$, ${\tilde{p}}_{i}$	Prediction distributions of the original and augmented samples, respectively.
$D_{KL}\left(\cdot ∥\cdot \right)$	Kullback–Leibler divergence used to measure prediction inconsistency between original and augmented samples.
$L_{con}$	Cross-modal consistency loss between the prediction distributions of original and augmented samples.
$L_{cls}$	Classification loss for cognitive bias recognition and psychological risk prediction.
$\lambda_{1}$, $\lambda_{2}$	Weighting coefficients controlling the contributions of semantic and cross-modal consistency losses.
L	Overall training objective combining classification and augmentation consistency losses.
$X_{t}$	Input text tensor containing cleaned and standardized student textual representations.
$H_{t}$, $W_{t}$, $C_{t}$	Text-sequence height, token embedding width, and number of semantic channels, respectively.
$L_{c}$, $L_{r}$, $L_{d}$	Numbers of contextual semantic encoding, theoretical rule adaptation, and cognitive bias discrimination layers, respectively.
$D_{t}$, $D_{h}$	Unified textual hidden width and intermediate feed-forward hidden width, respectively.
$M_{t}$	Number of attention heads in the contextual semantic encoder.
$Q_{t}$, $K_{t}$, $V_{t}$	Query, key, and value representations in the textual self-attention mechanism, respectively.
$A_{t}$	Self-attention weight matrix for textual contextual encoding.
$R_{t}$	Context-enhanced textual representation.
$C_{b}$	Theoretical cognitive bias prototype matrix constructed according to psychological theories.
$C_{b,k}$	Prototype representation of the *k*-th cognitive bias category.
$E_{i,k}$	Matching score between the *i*-th textual segment and the *k*-th cognitive bias prototype.
$\pi_{i,k}$	Theoretical affiliation weight between the *i*-th textual segment and the *k*-th cognitive bias prototype.
η	Theoretical enhancement coefficient.
${\hat{R}}_{t,i}$	Theory-enhanced representation of the *i*-th textual segment.
$z_{t,i}$	Global textual cognitive bias representation.
$L_{m}$	Inter-class margin loss for cognitive bias discrimination.
d(·,·)	Distance function between textual representations and theoretical prototypes.
Δ	Margin parameter for separating different cognitive bias categories.
$X_{b}$	Processed behavioral input tensor containing multichannel student learning behavior signals.
$H_{b}$, $W_{b}$, $C_{\mathrm{beh}}$	Learning-cycle length (number of time windows), behavioral feature width within each time window, and number of behavioral channels, respectively.
$L_{s}$, $L_{g}$, $L_{a}$	Numbers of local temporal convolution, gated recurrent state, and trend-sensitive attention layers, respectively.
$F_{b}$	Embedded behavioral feature representation in the unified hidden space.
$D_{b}$	Hidden representation width of the behavioral feature encoder.
$K_{b}$	Temporal kernel size of the one-dimensional causal convolution.
$Y_{t}^{\left(l\right)}$	Output representation of the *l*-th temporal convolution layer at time window *t*.
$\Theta_{r}^{\left(l\right)}$, $a^{\left(l\right)}$	Convolution parameter at temporal offset *r* and bias term of the *l*-th temporal convolution layer, respectively.
ψ(·)	Nonlinear activation function used in the temporal convolution layers.
$s_{t}$	Latent learning state representation at the *t*-th time window.
$\rho_{t}$	Update gate controlling the incorporation of new behavioral information into the learning state.
$\omega_{t}$	Reset/retention gate controlling the contribution of historical states to the candidate state.
${\bar{s}}_{t}$	Candidate learning state generated from the current behavioral representation and historical state.
$W_{\rho }$, $U_{\rho }$, $c_{\rho }$	Learnable parameters of the update gate.
$W_{\omega }$, $U_{\omega }$, $c_{\omega }$	Learnable parameters of the reset/retention gate.
$W_{s}$, $U_{s}$, $c_{s}$	Learnable parameters used to generate the candidate learning state.
$\delta_{t}$	Magnitude of behavioral state variation between two adjacent time windows.
$\mu_{t}$	Trend-sensitive attention weight assigned to the *t*-th learning state.
*q*	Learnable attention query vector used to calculate the importance of temporal states.
$W_{\mu }$, $V_{\mu }$	Learnable transformation matrices for the learning state and its temporal variation in the trend-sensitive attention mechanism.
$z_{b}$	Global temporal behavioral state representation obtained by attention-weighted aggregation of learning states.
$z_{i}$, $z_{s}$	Input representations of the classroom interaction and psychological scale modalities, respectively.
$D_{i}$, $D_{s}$	Feature widths of the classroom interaction and psychological scale modalities, respectively.
$L_{m}$	Number of layers in the modality projection network.
$D_{f}$	Unified feature dimension of different modalities after modality-specific projection.
$h_{m}^{\left(l\right)}$	Hidden representation of the *m*-th modality at the *l*-th projection layer.
$W_{m}^{\left(l\right)}$, $b_{m}^{\left(l\right)}$	Learnable weight matrix and bias vector of the *l*-th projection layer for the *m*-th modality.
$D_{m}^{\left(l\right)}$	Hidden feature width of the *m*-th modality at the *l*-th projection layer.
$h_{m}$	Projected representation of the *m*-th modality in the unified feature space.
$q_{m}$	Quality-description vector of the *m*-th modality, encoding factors such as missingness, temporal continuity, noise level, and sample coverage.
$D_{q}$	Dimension of the modality quality-description vector.
$\rho_{m}$	Estimated reliability score of the *m*-th modality.
$u_{r}$, $W_{r}$, $b_{r}$	Learnable parameters of the modality reliability estimation network.
M	Set of available modalities for the current sample.
$\lambda_{m}$	Reliability-modulated fusion weight assigned to the *m*-th modality.
$u_{f}$, $W_{f}$, $b_{f}$	Learnable parameters used to estimate task relevance for multimodal fusion.
$e_{m}$	Task-relevance score of the *m*-th modality used in the reliability-modulated fusion mechanism.
$z_{f}$	Final reliability-aware cross-modal fused representation.
$L_{o}$	Number of layers in the multitask output network.
${\hat{y}}_{c}$	Predicted probability distribution over cognitive bias categories.
${\hat{y}}_{r}$	Predicted probability distribution over psychological risk levels.
$W_{c}$, $b_{c}$	Learnable parameters of the cognitive bias classification output layer.
$J_{c}$	Loss function for cognitive bias recognition.
$J_{r}$	Loss function for psychological risk prediction.
α, β	Weighting coefficients controlling the contributions of psychological risk prediction and cross-modal consistency regularization, respectively.
J	Overall multitask optimization objective of the reliability-aware cross-modal fusion module.

**Table 4:** Training configuration and hyperparameter settings.

Parameter	Value
Maximum textual input length	128
Batch size	32
Number of training epochs	50
Optimizer	AdamW
Initial learning rate	$2\times 10^{-5}$
Weight decay coefficient	$1\times 10^{-4}$
Dropout rate	0.3
Behavioral temporal encoding hidden dimension	128
Multimodal fusion representation dimension	256
Number of attention heads	4
Task loss weighting strategy	Equal weighting
$\lambda_{1}$	0.5
$\lambda_{2}$	0.5
Early stopping patience	10 epochs
Class imbalance handling	Class-weighted loss
Random seed strategy	Fixed random seeds

**Table 5:** Performance comparison of different methods on cognitive bias recognition. Bold values indicate the best performance achieved among all compared methods for each evaluation metric.

Method	Accuracy	Precision	Recall	Macro-F1	Conf.
Logistic Regression	0.684±0.014	0.661±0.016	0.648±0.017	0.653±0.015	0.712±0.013
SVM	0.701±0.013	0.682±0.015	0.667±0.016	0.674±0.014	0.724±0.012
Random Forest	0.719±0.012	0.701±0.014	0.684±0.015	0.691±0.013	0.738±0.012
XGBoost	0.736±0.011	0.718±0.013	0.703±0.014	0.710±0.012	0.751±0.011
TextCNN	0.758±0.010	0.741±0.012	0.726±0.013	0.733±0.011	0.769±0.010
BiLSTM	0.771±0.010	0.754±0.011	0.739±0.012	0.746±0.011	0.781±0.010
BERT	0.812±0.009	0.798±0.010	0.786±0.011	0.791±0.010	0.824±0.009
Behavior-Transformer	0.783±0.010	0.766±0.011	0.752±0.012	0.759±0.011	0.796±0.010
Multimodal Transformer	0.834±0.008	0.821±0.009	0.809±0.010	0.814±0.009	0.846±0.008
BCL	0.851±0.007	0.838±0.008	0.825±0.011	0.837±0.009	0.862±0.007
shi2025	0.849±0.006	0.840±0.011	0.830±0.009	0.839±0.008	0.863±0.009
**MLBS-Net**	0.872±0.006	0.861±0.007	0.849±0.008	0.855±0.007	0.889±0.006

**Table 6:** Performance comparison of different methods on psychological risk early warning. Bold values indicate the best performance achieved among all compared methods for each evaluation metric.

Method	AUC	PR-AUC	Recall	F1-Score	Conf.
Logistic Regression	0.721±0.015	0.684±0.017	0.642±0.018	0.659±0.016	0.703±0.014
SVM	0.736±0.014	0.701±0.016	0.658±0.017	0.674±0.015	0.718±0.013
Random Forest	0.758±0.013	0.724±0.015	0.681±0.016	0.696±0.014	0.739±0.012
XGBoost	0.779±0.012	0.746±0.014	0.704±0.015	0.718±0.013	0.756±0.012
TextCNN	0.742±0.014	0.711±0.016	0.672±0.017	0.687±0.015	0.728±0.013
BiLSTM	0.768±0.013	0.735±0.015	0.695±0.016	0.709±0.014	0.747±0.012
BERT	0.801±0.011	0.772±0.013	0.731±0.014	0.748±0.012	0.793±0.011
Behavior-Transformer	0.824±0.010	0.796±0.012	0.759±0.013	0.773±0.011	0.815±0.010
Multimodal Transformer	0.851±0.009	0.823±0.011	0.786±0.012	0.802±0.010	0.842±0.009
BCL	0.872±0.008	0.840±0.007	0.807±0.011	0.827±0.007	0.862±0.009
shi2025	0.874±0.009	0.843±0.011	0.805±0.009	0.828±0.008	0.860±0.006
**MLBS-Net**	0.891±0.006	0.867±0.008	0.828±0.009	0.841±0.008	0.884±0.006

**Table 7:** Ablation study of different components in MLBS-Net. Bold values indicate the best performance achieved among all compared methods for each evaluation metric.

Variant	Acc.	Macro-F1	AUC	PR-AUC	Conf.
*w*/*o* Theory-Guided Textual Encoding	0.831±0.009	0.808±0.010	0.857±0.009	0.829±0.011	0.842±0.009
*w*/*o* Temporal Behavior Modeling	0.846±0.008	0.821±0.010	0.839±0.010	0.812±0.012	0.826±0.010
*w*/*o* Reliability Estimation	0.852±0.008	0.827±0.009	0.861±0.009	0.835±0.010	0.831±0.009
*w*/*o* Cross-Modal Interaction	0.838±0.009	0.815±0.010	0.849±0.010	0.821±0.011	0.817±0.010
*w*/*o* Multitask Learning	0.844±0.008	0.819±0.010	0.852±0.009	0.826±0.011	0.829±0.009
*w*/*o* Theory Consistency Constraint	0.856±0.008	0.832±0.009	0.866±0.008	0.841±0.010	0.846±0.008
*w*/*o* Quality Cue Input	0.849±0.008	0.824±0.009	0.858±0.009	0.833±0.010	0.822±0.009
*w*/*o* Teacher Feedback Branch	0.861±0.007	0.839±0.008	0.872±0.008	0.847±0.009	0.851±0.008
Text + Behavior Only	0.854±0.008	0.831±0.009	0.869±0.008	0.844±0.010	0.849±0.008
**Full MLBS-Net**	0.872±0.006	0.855±0.007	0.891±0.006	0.867±0.008	0.884±0.006

**Table 8:** Computational complexity comparison of different models.

Model	Parameters (M)	FLOPs (G)	Inference Time (ms/Sample)	GPU Memory (GB)
Logistic Regression	0.023	0.0013	0.083	0.152
SVM	0.047	0.0037	0.362	0.217
Random Forest	1.184	0.0116	0.647	0.328
XGBoost	3.672	0.0234	0.873	0.463
TextCNN	3.264	0.462	1.927	0.934
BiLSTM	8.743	1.284	3.684	1.673
BERT	109.84	22.73	18.92	4.936
Behavior Transformer	28.76	6.947	9.386	2.846
Multimodal Transformer	86.42	19.27	16.84	5.738
MLBS-Net	15.93	3.576	5.847	1.973

## Data Availability

The data presented in this study are available upon request from the corresponding author.
